# Synergistic Effect of Paclitaxel and Epirubicin Coadministration—Insight into the Mechanisms of Interactions with Model Breast Cancer Cell Membranes

**DOI:** 10.1021/acs.langmuir.5c02558

**Published:** 2025-09-29

**Authors:** Damian Dziubak, Paulina Kaczmarczyk, Izabella Leszczyńska, Piotr Batys, Philippe Fontaine, Dorota Matyszewska

**Affiliations:** † Faculty of Chemistry, Biological and Chemical Research Centre, 49605 University of Warsaw, Żwirki i Wigury 101, 02089 Warsaw, Poland; ‡ Jerzy Haber Institute of Catalysis and Surface Chemistry, Polish Academy of Sciences, Niezapominajek 8, 30239 Krakow, Poland; § Synchrotron Soleil, L’Orme des Merisiers, Départementale 128, 91190 Saint-Aubin, France

## Abstract

This study investigates the synergistic interaction mechanism of paclitaxel (PTX) and epirubicin (EPI) coadministration with model breast cancer cell membranes formed by monocomponent and ternary phospholipid monolayers composed of DPPC, cholesterol, and DMPS (4:4:2 molar ratio) at the air–water interface. These biomimetic membrane models were characterized using a combination of interface-sensitive techniques, including Langmuir monolayers, compression–expansion cycles, grazing incidence X-ray diffraction (GIXD), quartz crystal microbalance (QCM), and attenuated total reflection spectroscopy (ATR) for supported layers. These experimental methods were complemented by molecular dynamics (MD) simulations to gain molecular-level insights. The study confirms that neutral PTX interacts with all membrane components, while positively charged EPI exhibits significant interactions predominantly with the negatively charged DMPS lipid. Notably, the PTX + EPI combination demonstrated pronounced synergistic effects on both types of phospholipid monolayers, especially the ternary mixture, leading to significant membrane fluidization and the formation of irreversible aggregates. GIXD further corroborated the increased membrane fluidity and structural reorganization induced by the drug combination. QCM and ATR spectroscopy revealed substantial structural alterations and lipid rearrangement in the supported bilayers upon exposure to PTX + EPI. MD simulations suggest that these synergistic effects result from the formation of drug clusters within the lipid bilayer, influencing the physicochemical properties of the model biomembranes. These findings provide valuable insights into the interfacial interactions of anticancer drugs with lipid membrane materials, which can contribute to the development of improved combination therapies.

## Graphical abstract

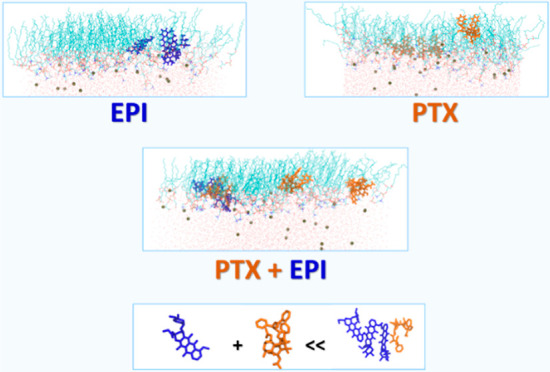

## 1 Introduction

According to data from the World Health Organization, 2.3 million women were diagnosed with breast cancer worldwide in 2020, and 685,000 died from the disease.^1^ Breast cancer occurs in every country and affects women of all ages, although the likelihood of developing the disease increases with age, making it the most common cancer diagnosis globally.^2^ For this reason, it is essential to explore new and effective anticancer strategies and to deepen research on existing drugs to optimize their application and improve treatment outcomes. Combination therapies are commonly used in breast cancer treatment. A typical regimen includes the administration of a taxane (e.g., docetaxel) in combination with an anthracycline antibiotic (e.g., doxorubicin), also followed by cyclophosphamide.^3,4^ However, the duration of this regimen is often long, delaying the start of radiotherapy and potentially reducing its effectiveness. It also requires multiple drug doses, increasing the likelihood of side effects and diminishing patients’ quality of life. Consequently, there has been growing interest in using alternative therapies, including epirubicin and paclitaxel as a combined therapy.^5,6^ Studies have shown that such combinations can reduce the risk of recurrence and the mortality of breast cancer. Despite these advancements, the challenges remain in anticancer therapy due to diagnostic limitations and the complexities of the disease.

Monolayers of lipids at the air–water interface are valuable simplified models of cancer cell membranes to study interactions with anthracycline drugs, such as doxorubicin and daunorubicin.^7–12^ This method allows precise control over membrane composition and packing density, which is essential for mimicking the unique lipid environments of cancerous cells.^13^ By adjusting the lipid constituents, researchers can simulate the altered membrane properties characteristic of cancer cells, providing insights into the mechanisms of action and efficacy of candidate drugs and therapies. It has been demonstrated that anthracyclines can intercalate into lipid monolayers, affecting membrane fluidity and stability. More specifically, doxorubicin and idarubicin interact differently with lipid monolayers, affecting their penetration and distribution within the membrane.^14^ The possibility of penetration of model membranes relies on the electrostatic interactions between the positively charged anthracyclines and negatively charged components of the model systems, although drug lipophilicity, steric hindrance, and ability to form dimers/polymers may also play a role.^9,15–17^

In their studies, Zhao and Feng examined the interaction of paclitaxel with monolayers composed of a mixture of dipalmitoylphosphatidylcholine (DPPC) and cholesterol in various molar ratios, simulating biological cell membranes.^18,19^ Analysis of the compression isotherms revealed that DPPC, paclitaxel, and cholesterol can form a nonideal, miscible system at the air–water interface. It was demonstrated that cholesterol enhances the intermolecular forces between DPPC and paclitaxel, thereby stabilizing the monolayer. Furthermore, studies on the penetration of paclitaxel into the DPPC/cholesterol mixture indicated that the presence of cholesterol significantly reduces the drug’s ability to penetrate the monolayer. Further detailed studies revealed that increasing cholesterol content alters monolayer packing, reduces the penetration and integration of paclitaxel into the lipid layer, highlighting cholesterol’s ability to modulate drug–membrane interactions.^18^ Such concentration-dependent effects of cholesterol on monolayer properties may have direct implications for cholesterol-rich environments, such as cancer cells or lipid rafts. This aspect was further explored utilizing Langmuir monolayers composed of DPPC, sphingomyelin (SM), and cholesterol in different ratios as simple models of lipid rafts.^20^ Similarly, the Langmuir monolayers were used by Preetha et al. in research comparing the penetration of paclitaxel through model cell membranes of healthy and cancerous cervical cells.^21^ The analysis of their results showed that paclitaxel more easily penetrates the monolayer mimicking the cell membrane of a healthy cell compared to that of a cancer cell. This difference was attributed to the higher levels of cholesterol and sphingomyelin in the membranes of cervical cancer cells, which increased their rigidity and reduced permeability.

In the studies mentioned above, the composition of the model membranes was either not adjusted to reflect the lipid composition of cancer cell membranes or reflected only in a very general manner. This task is inherently challenging because there is no single characteristic lipid profile that universally distinguishes cancer cells from noncancerous cells. The lipid composition of cancer cells varies significantly depending on the type of cancer.^22,23^ In cancer cells, lipid asymmetry undergoes substantial reorganization, leading to the exposure of negatively charged phosphatidylserine (PS) residues on the outer leaflet of the plasma membrane.^24^ This alteration is associated with an enhanced cell proliferation. Additionally, the content of cholesterol, a sterol which regulates membrane fluidity under physiological conditions, varies across different types of cancer.^23^ Often, a shift in the phosphatidylcholine-to-cholesterol (PC:Chol) ratio is observed, with an increase in the cholesterol content. However, this shift is cancer-type specific. For example, the lipid profile of breast cancer cells reveals an overall increase in the total phospholipid content compared to healthy breast cells, with a notable rise in PS levels.^25–27^ Simultaneously, breast cancer cells exhibit significantly reduced phosphatidylinositol (PI) levels.^28,29^ Furthermore, breast cancer cells are characterized by a relatively high cholesterol content, further influencing their membrane properties.

The model phospholipid membranes and the changes in their surface properties upon exposure to drugs can be studied using numerous experimental techniques. Effect of drugs on phospholipid monolayers formed at the air–water interface may be followed by neutron reflectometry^14,30^ or grazing incidence X-ray diffraction,^9,31^ which provide information on the drug location within the model membrane and the changes in its organization. Supported phospholipid bilayers may provide a better model of biological membranes. Additionally, the transfer of such layers onto a solid support allows one to employ electrochemical or spectroscopic techniques to follow the changes in the membrane homogeneity and permeability, as well as its structure upon exposure to drugs. Supported phospholipid bilayers have been used to study the effect of anthracyclines using this approach.^32–36^

Apart from experimental methods, molecular dynamics (MD) simulation is also a widely used tool in pharmaceutical research to investigate the interactions between drugs and lipid membranes.^37^ MD simulations have mainly focused on the behavior of molecules in membranes, affecting membrane structure and properties.^38^ Additionally, the transport of drugs through lipid membranes has been also intensively investigated.^39,40^ Previous MD studies provided better understanding of anthracycline drug–membrane interactions for doxorubicin.^41^ The simulations allowed exploration of its interaction with membranes having different lipid compositions^41,42^ providing information on how they vary with changes in lipid type and packing density, which directly impacts drug delivery efficiency.^43^ Additionally, MD simulations enable revealing the transfer mechanisms, aggregation, and structural changes of common chemotherapeutic drugs, such as doxorubicin, in phospholipid membranes.^44,45^

In this work, we aim to precisely compare the impact of two types of drugs used in anticancer therapy: paclitaxel and epirubicin (Figure 1) separately and in combination on model phospholipid membranes prepared at the air–water interface and further supported on a solid support. The novelty of this study relies on the specific composition of the model systems, which was carefully designed based on the available literature data to reflect the main components of the cell membranes of breast cancer cells both in terms of the polar headgroup composition and the length and saturation of acyl chains, which may apparently contain slightly elevated levels of longer saturated (16:0) acyl chains.^25,28,29^ Therefore, the model membranes consisted of the representative of phosphatidylcholines (DPPC) as one of the major components of eukaryotic cell membranes, phosphatidylserines (DMPS) as typical markers of cancer changes within membrane lipidomics, and cholesterol (Figure 1), which is responsible for regulating membrane fluidity and the levels of which may significantly change during the development of cancer. The interactions of both individual drugs and their mixture were examined, and their impact on the physicochemical properties of mono- and three-component phospholipid layers formed at the water–air interface was determined in situ using thermodynamical studies supplemented with grazing incidence X-ray diffraction (GIXD) results providing information on the 2D organization of the molecules within the monolayer. Additional information regarding the interactions was also obtained for bilayers transferred onto a solid support, which were analyzed using attenuated total reflectance spectroscopy (ATR) and quartz crystal microbalance with dissipation (QCM-D). The results of the experimental studies were complimented with the conclusions on the mechanism of action of the drugs with phospholipid membranes, obtained based on MD simulations.

**Figure 1:**
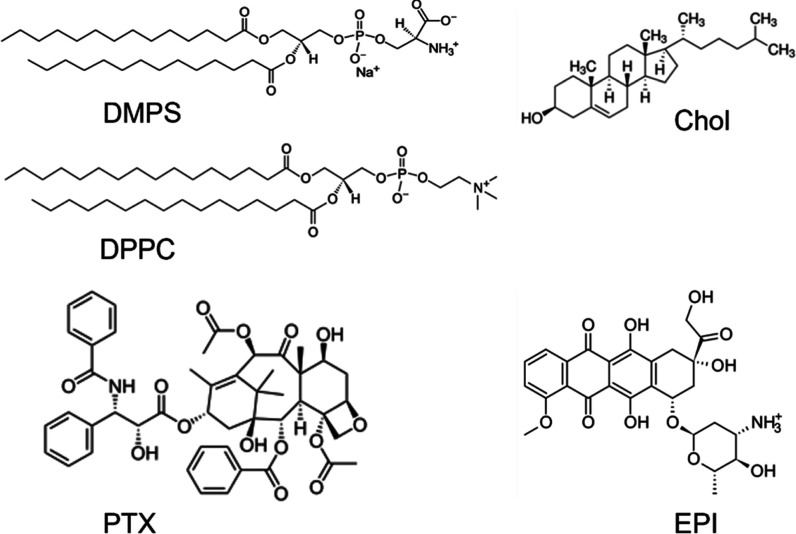
Structures of components of the model breast cancer membranes: DMPS, DPPC, and cholesterol (Chol) and anticancer agents: paclitaxel (PTX) and epirubicin (EPI).

## 2 Experimental Section

### 2.1 Materials

1,2-Dipalmitoyl-*sn*-glycero-3-phosphocholine (DPPC) and 1,2-dimyristoyl-*sn*-glycero-3-phospho-l-serine (sodium salt) (DMPS) were purchased from Avanti Polar Lipids (USA). Cholesterol, organic solvents: chloroform and methanol (HPLC grade), paclitaxel (PTX), and epirubicin (EPI) were obtained from Merck (Poland). Lipid solutions (0.5–1 mg/mL) were prepared in a chloroform:methanol 9:1 v/v mixture. The mixed DPPC:Chol:DMPS 4:4:2 solutions were prepared by mixing the appropriate volumes of stock solutions of individual components to obtain the desired molar ratio. Milli-Q water of the resistivity 18.2 MΩ·cm (Millipore) was used throughout Langmuir, GIXD, and QCM-D experiments, while D_2_O was used for ATR measurements.

### 2.2 Methods

#### 2.2.1 Langmuir Method

A KSV NIMA (Biolin, Sweden) Langmuir trough (medium size with a total area of 243 cm^2^) with a Wilhelmy microbalance equipped with a sensor made of Whatman filter paper and two hydrophilic barriers was employed. Prior to the experiment, the trough was cleaned with methanol, chloroform, and Milli-Q water, and next, the appropriate volumes of lipid solutions were deposited dropwise at the air–water interface by a Hamilton microsyringe. After 10 min of solvent evaporation, the compression of the barriers with a speed of 10 mm/min was started to register surface pressure (±0.1 mN m^–1^) versus mean molecular area. The monolayers were formed either on a pure Milli-Q water subphase or subphase containing PTX or EPI solutions (10^–6^ mol/L) as well as the mixture of PTX and EPI with the concentration of 10^–6^ mol/L each. The concentration of the drugs was chosen based on previously published results on drug–lipid interactions to allow the comparisons and was also determined by the upper limit of water-solubility of PTX. All experiments were carried out at 21 °C, and the results shown in the figures and reported in the tables represent the average of at least three measurements.

Based on Langmuir isotherms, it is possible to calculate compression modulus (*C*_s_^–1^), which is defined as

1 $$C_{s}^{-1}=-A{\left(\frac{d\pi }{dA}\right)}_{T,N}$$

It provides information about the elastic properties of the layers and phases, in which a monolayer exists. The values of *C*_s_^–1^ correspond to the phase of the monolayer such as gas phase (G, 0–12.5), liquid-expanded phase (LE, 12.5–100 mN m^–1^), liquid-condensed phase (LC, 100–250 mN m^–1^), and solid phase (S, >250 mN m^–1^).^46,47^ Additionally, the minima on the compression modulus versus surface pressure plot provide information on the phase transitions or collapse of the monolayer.^48,49^

In case of mixed monolayers, the forces between the components may be compared based on the parameters calculated from the isotherms of single- and multicomponent monolayers.^50,51^ The theoretical area of the mixed layer (*A*_1...*N*_^id^) and the excess area (*A* ^Exc^) are defined as follows:

2 $$A_{1...N}^{id}=\sum_{N}^{1}A_{i}X_{i}$$

3 $$A^{Exc}=A_{1...N}-A_{1...N}^{id}$$

where *A*_*i*_ is the area of the monolayers of the individual components at the selected surface pressure and *X*_*i*_ is the molar ratio of the single component in the mixed layer. *A* ^Exc^ denotes the difference between the area per molecule of mixed monolayer (*A*_1...N_) at the selected surface pressure and the theoretical area per molecule (*A*_1...*N*_^id^) calculated from eq 2.

The possible synergistic effect of the two drugs applied together (Δ*A*_PTX+EPI_) was determined based on the changes in the area per molecule at selected surface pressures (Δ*A*_PTX_ or Δ*A*_EPI_) obtained from the isotherms recorded in the presence (*A*_PTX_ or *A*_EPI_) and absence ( $A_{H_{2}O}$ ) of individual drugs in the subphase (PTX and EPI):^52^

4 $$\Delta A_{PTX}=A_{PTX}-A_{H_{2}O}$$

5 $$\Delta A_{EPI}=A_{EPI}-A_{H_{2}O}$$

6 $$\Delta A_{PTX+EPI}=A_{PTX+EPI}-A_{H_{2}O}$$

If the difference in the area per molecule in the presence of the drug mixture (Δ*A*_PTX+EPI_) is greater than the sum of the differences in the area per molecule calculated for the individual drugs (Δ*A*_PTX_ + Δ*A*_EPI_), the synergy is indeed observed.^52^

In order to investigate the effect of anticancer agents dissolved in the subphase on the already formed model cancer cell membranes, the mixed monolayers were first compressed to the biologically relevant value of 30 mN m^–1^, at which the organization and surface properties of monolayers resemble those of bilayers.^53,54^ After this target pressure was reached and the barriers were stopped, stock solutions of anticancer drugs were injected underneath the compressed monolayers to obtain the final drug concentration in the subphase of 10^–6^ mol/L. Then changes of surface pressure in time were recorded.

The reversibility of the model systems was probed by recording multiple compression–expansion cycles in the surface pressure range from 0 to 30 mN m^–1^. Based on the first cycle, three main thermodynamic parameters were calculated: the free energy (Δ*G* ^hys^), the entropy (Δ*S* ^hys^), and the enthalpy (Δ*H* ^hys^) of hysteresis:^55–57^

7 $${\Delta G}_{comp/\exp}=N_{A}\int_{1mN/m}^{30mN/m}A\;d\pi$$

8 $$\Delta G^{hys}={\Delta G}_{\exp}-{\Delta G}_{comp}$$

9 $${\left[{\Delta S}_{\pi }^{hys}=R\;\ln\frac{A_{\exp}}{A_{comp}}\right]}_{\pi }$$

10 $${\Delta S}^{hys}=\sum_{\pi }\Delta S_{\pi }^{hys}$$

11 $${\Delta H}^{hys}={\Delta G}^{hys}+{T\Delta S}^{hys}$$

where *A*_comp_, *A*_exp_, and Δ*G*_comp_, Δ*G*_exp_ are the area and energy of the compression and expansion, respectively, *N*_A_ is the Avogadro number, and *R* is the gas constant.

#### 2.2.2 Grazing Incidence X-ray Diffraction (GIXD)

In order to probe the organization of the phospholipids at the air–water interface, the GIXD experiments were performed at the SIRIUS beamline in SOLEIL synchrotron (Gif-sur-Yvette, France).^58^ The construction of the diffractometer as well as the working parameters of the synchrotron beam can be found on the SOLEIL Web site (www.synchrotron-soleil.fr) and in the review articles together with the principles of the method.^59,60^ The setup consists of the liquid surface diffractometer and the Langmuir trough (R&K GmbH Electronics, Germany). It is placed in a gastight box flushed with helium, which is meant to reduce background scattering and possible sample damage. The scattered signal coming from the incoming X-ray beam with the energy of 8 keV (λ = 1.55 Å) was detected by a Pilatus3 2D pixel detector (Dectris Ltd., Switzerland) associated with a Soller collimator (JJ X-ray Denmark) of angular resolution 0.07. Prior to the measurement, the phospholipid monolayers were compressed to 30 mN m^–1^ (surface pressure held constant during the measurement) and the patterns were collected with the resolution of approximately 0.005 Å^–1^ by scanning the in-plane 2θ angle. As a result, the intensity maps *I*(*Q*_*xy*_,*Q*_*z*_) were obtained, where *Q*_*xy*_ and *Q*_*z*_ are the components of the scattering vector, 2θ_*XY*_ is the angle between the incident and diffracted beams projected onto the horizontal plane, and α_f_ represents the beam exit angle:^59,61–63^

12 $$Q_{XY}=\frac{4\pi }{\lambda }\;\sin\left(\frac{2\theta_{XY}}{2}\right)$$

13 $$Q_{Z}=\frac{2\pi }{\lambda }\;\sin\left(\alpha_{f}\right)$$

The *I*(*Q*_*xy*_) dependence and the occurring peaks allow for the determination of the crystalline phases and unit cell parameters, while the information on the molecular tilt of the molecules forming the monolayer at the air–water interface may be derived from the *Q*_*z*_ parameter. The cell parameters can be calculated using the following formula:^64^

14 $$d_{spacing}=\frac{2\pi }{Q_{XY}}={\left[\frac{h^{2}}{a^{2}}+\frac{k^{2}}{b^{2}}-2\left(\frac{hk}{ab}\right)\;\cos\gamma \right]}^{-1/2}\;\sin\left(\gamma \right)$$

where *d*_spacing_ is the repeat distance in the 2D lattice, *a* and *b* are lattice parameters derived from the position of the Bragg peaks’ maxima, *h* and *k* denote Miller indices, and γ is the angle between the lattice vectors. Additionally, the tilt angle (τ) pointing the deviation of the orientation of the molecules from a straight line perpendicular to the surface of the subphase is defined by the following equation:^64^

15 $$Q_{Z}=Q_{XY}\;\tan\left(\tau \right)\;\cos\psi$$

where ψ is the angle between the *Q*_*XY*_ vector and the tilt direction. Another parameter is the area of a unit cell (*A*_uc_), containing one tail for the hexagonal unit cells and two tails for the rectangular unit cells:

16 $$A_{uc}=ab\;\sin\left(\gamma \right)$$

The full width at half-maximum (fwhm) of the dependence of the intensity on *Q*_*XY*_ is used to calculate the in-plane coherence length (*L*_*XY*_), which provides information on the range of 2D crystallinities:^65^

17 $$L_{XY}=\frac{2}{{fwhm}_{Q_{XY}}}$$

#### 2.2.3 Molecular Dynamics Simulations

The all-atom molecular dynamics simulations of the lipid monolayer were performed by GROMACS software, version 2022.3.^66^ The CHARMM force field was employed because of suitability with drug–membrane interactions and an extensive validation.^67,68^ The explicit TIP3P model of water was applied to solvating the systems.^69^ Parameters and topology files for paclitaxel (PTX) and epirubicin (EPI) were generated by CHARMM-GUI Ligand Reader & Modeler.^70^ Then, the CHARMM-GUI Web server was used to generate a monolayer consisting of DPPC, cholesterol (Chol), and DMPS at a molar ratio 4:4:2, matching the experimentally studied membranes. In total, four systems were investigated, each consisting of monolayers with 80, 80, and 40 molecules of DPPC, Chol, and DMPS, respectively. The simulation box dimension was 8 × 8 × 25.5 nm^3^. The first two simulation sets (referred to as “single drug”) consisted of 6 PTX or 6 EPI molecules. The third system (referred to as a “drug mixture”) was composed of 12 molecules: 6 EPI and 6 PTX. As a reference, we also simulated the pure membrane. Periodic boundary conditions were applied in all directions. The Lennard-Jones potential with a 1 nm cutoff was used to describe van der Waals interactions between atoms. Bonds between hydrogen and heavy atoms were constrained by the LINCS algorithm^71^ and water by the SETTLE algorithm.^72^ The long-range electrostatic interactions were calculated by the Ewald method with a Fourier grid spacing of 0.12 nm. The simulations were run under constant conditions of temperature and volume (*NVT* ensemble). A temperature of 294 K was controlled by a V-rescale thermostat^73^ with a coupling time constant of 5 ps. The simulation was run for a total duration of 300 ns. The first 50 ns of simulation corresponds to the equilibration and was disregarded from analysis. All molecular visualizations employed the VMD software.^74^

The radial distribution function (RDF) was calculated using the built-in GROMACS tool *gmx rdf*. RDF describes how the density of molecules changes as a function of distance from a chosen reference molecule. It provides information about the structure of the system, e.g., whether the molecules cluster at specific distances in an ordered manner (peaks in *g*(*r*) > 1) or are uniformly distributed (peak values of *g*(*r*) = 1). In the performed simulations, the reference was either EPI or PTX center of mass.

Hydrogen bonding, calculated using the built-in Gromacs tool *gmx hbond*, was assessed based on the geometric criteria for which the acceptor–donor distance was less than 0.35 nm and the H-bond angle was less than 30°.

#### 2.2.4 Quartz Crystal Microbalance with Dissipation (QCM-D)

Vibration frequency and energy dissipation in response to the influence of the drug on the model lipid membrane were monitored using a quartz crystal microbalance with a dissipation monitoring (QCM-D) technique using the QSense E1 instrument (Q-Sense AB, Sweden). A silicon dioxide sensor (QS QSX303) was used as the substrate. Before the experiment, the prism was cleaned by immersion in a 5% SDS solution, followed by sonication. Subsequently, the sensor was thoroughly rinsed with ultrapure water and dried under an UV lamp. The phospholipid membrane was prepared using the rapid solvent exchange method.^75^ Initially, the cleaned substrate was immersed in a solution containing dissolved lipids in a mixture of organic solvents followed by multiple rinses with water to ensure complete removal of the organic solvent. The sensor with the reconstituted membrane was then mounted in a flow cell. Water was introduced into the membrane-coated sensor measurement cell, and the system was allowed to stabilize for approximately 20 min. After stabilization, the drug solution (10^–6^ mol/L PTX or 10^–6^ mol/L EPI) or drug mixture (10^–6^ mol/L PTX + EPI each) was introduced.

#### 2.2.5 Attenuated Total Reflectance Spectroscopy (ATR)

For infrared measurements, the attenuated total reflectance (ATR) method was used, utilizing a Nicolet iS50 FTIR spectrometer (Thermo Fisher Scientific, Waltham, MA, USA) equipped with an MCT-A detector cooled by liquid nitrogen and a custom single-reflection accessory. Before use, the prism was polished with diamond suspensions of varying particle sizes (0.5, 1, and 3 μm) on polishing cloths. Following this, the substrate was rinsed with ultrapure water and dried. Next, 1 mL of 2% HF was dropped and left for 5 min to etch the silicon surface. After rinsing with water, the substrate was placed in ethanol for 30 min in an ultrasonic bath. The substrate was then modified with APTES ((3-aminopropyl)­triethoxysilane) and mounted in an IR cell. The membrane was formed using the rapid solvent exchange method.^75^ First, a background spectrum (*I*_0_) was collected with the clean prism surface in D_2_O. The D_2_O was removed, and 1 mL of a 10^–3^ mol/L lipid mixture solution was applied to the prism surface. After 5 min, the surface was gently washed with multiple amounts of D_2_O to ensure complete removal of the organic solvent, following the collection of the membrane spectrum in D_2_O (*I*_1_). The absorbance spectrum was then plotted as

18 $$A=\log\frac{I_{0}}{I_{1}}$$

versus wavenumber. After 30 min, the stability of the membrane was verified by checking if there were any spectral changes. Upon confirming membrane stability, a drug stock solution was added to the cell to achieve a final concentration of 10^–6^ mol/L, and the spectrum (*I*_2_) was collected 120 min post-drug addition. The resulting spectrum was plotted as

19 $$A=\log\frac{I_{0}}{I_{2}}$$

versus wavenumber. Additionally, to determine the tilt angle of the hydrocarbon chains relative to the surface normal, spectra were collected at IR beam polarizations of 0 and 90°. The angles were calculated using the formulas provided in the Supporting Information.

## 3 Results and Discussion

### 3.1 Single-Component Model Membranes at the Air–Water Interface

Phospholipid monolayers of single components formed at the air–water interface using the Langmuir technique were first employed to study the effect of PTX and EPI applied individually and subsequently as a drug mixture. The drugs were dissolved in the subphase, on which the phospholipid monolayers were formed and the surface pressure–area per molecule isotherms (π–*A*) were recorded (Figure 2).

**Figure 2:**
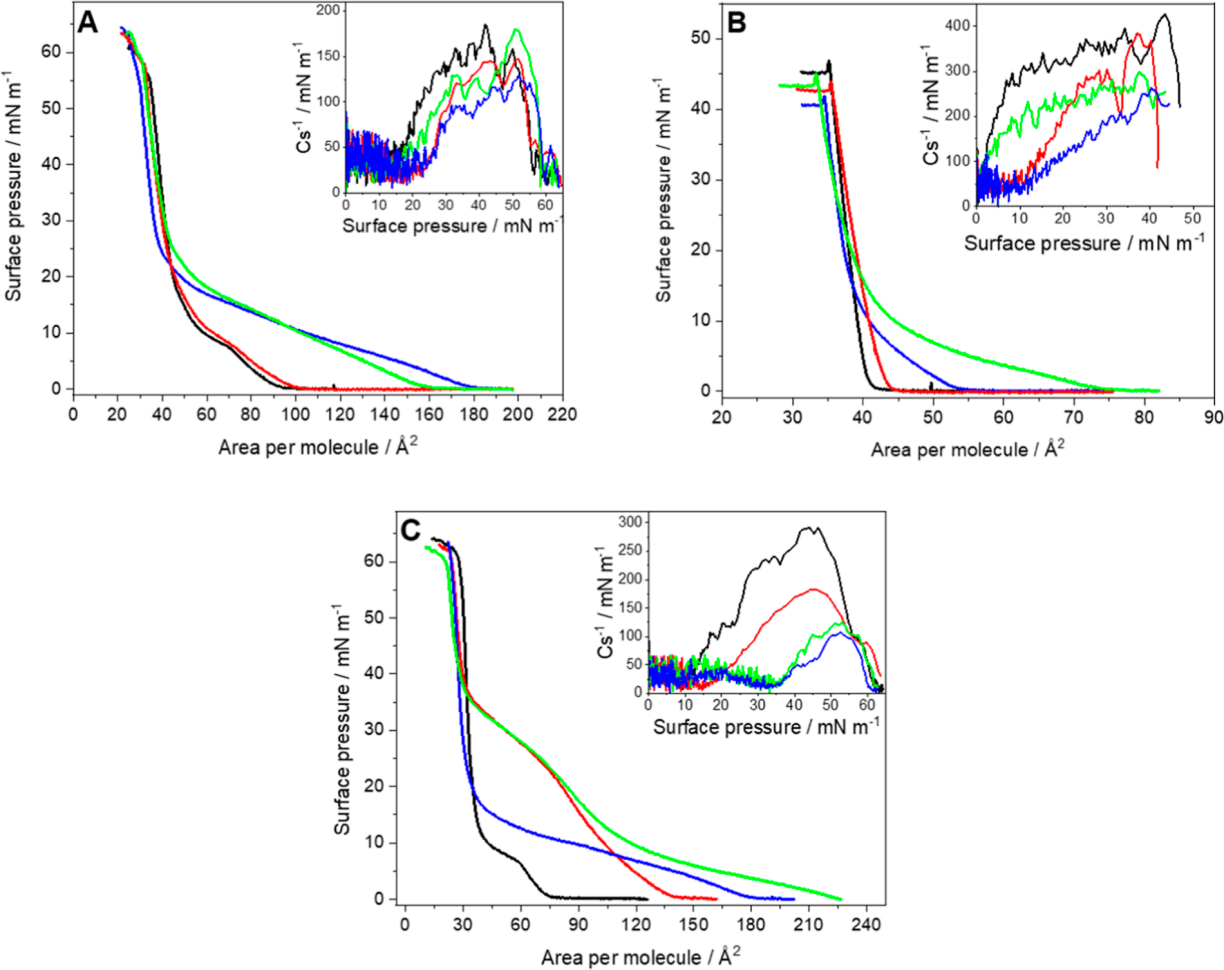
Surface pressure–area per molecule (π–*A*) isotherms of (A) DPPC, (B) cholesterol, and (C) DMPS monolayers on the pure water subphase (black) and the water subphase containing 10^–6^ mol/L PTX (blue), 10^–6^ mol/L EPI (red), and 10^–6^ mol/L PTX + EPI (green). Inset: compression modulus vs surface pressure plot. (*T* = 21 ± 1 °C).

The influence of individual drugs on the single components of the model breast cancer cell membrane strongly depends on both the properties of the phospholipid and the drug itself. Epirubicin, similarly to other anthracyclines with relatively low lipophilicity, shows very limited interactions with phosphatidylcholines such as DPPC (Figure 2A) at this concentration. Similarly, the influence on cholesterol monolayers exposed to EPI (Figure 2B) is also small, but more pronounced, especially in terms of the reduction of the maximum value of the compression modulus (Table S1).^14,76,77^ Apparently, even small amounts of EPI interacting with cholesterol at the air–water interface are able to influence the organization of cholesterol molecules without causing a significant shift of the isotherm. However, the influence on monolayers of DMPS is very clear due to the presence of electrostatic attractions between the negatively charged polar head of this lipid and positively charged anthracycline (Figure 2C).^7,14^ It results in a shift of the LE–LC transition plateau from ca. 10 mN m^–1^ to 30 mN m^–1^ and a shift of the lift-off area from 70 to 130 Å^2^ per molecule. These features prove the incorporation of the drug into the phospholipid membrane and the strong interaction of the drug with this phospholipid. It also leads to the significant decrease in the maximum value of compression modulus, which shows that the monolayer changes its phase from solid to liquid-condensed, since the *C*_s_^–1^ value for the DMPS monolayer in the presence of EPI is close to the border value of the LE–LC phase (Table S1).^46,47^ Further compression of the DMPS monolayer in the presence of EPI results in the expulsion of the drug from the layer, as shown by the isotherms showing lower values of areas per molecule in the presence of EPI compared to the pure subphase at surface pressures higher than 40 mN m^–1^.

Different situations are observed for PTX interactions with single-component monolayers at the air–water interface (Figure 2). The drug incorporates into DPPC monolayers leading to the significant increase in the lift-off area but seems to be expelled from the layer upon further compression. A similar situation is observed for cholesterol monolayers; in the presence of PTX in the subphase, the lift-off area of the isotherm is shifted toward larger areas per molecule, but at the surface pressure of approximately 15 mN m^–1^, the isotherm converges with the Chol isotherm on the pure water subphase, which again suggests that the drug is no longer present in the layer at the air–water interface. The effect of PTX on DMPS monolayers is comparable to the effect on DPPC monolayers. The lift-off area is shifted to greater values, which suggests the partitioning of the drug in the monolayer and the plateau region typically observed for the DMPS monolayer is not that well-developed in the presence of PTX in the subphase. Further compression of the DMPS monolayer again leads to the expulsion of the drug, and above approximately 15 mN m^–1^, the isotherm in the presence of PTX converges with the isotherm of DMPS formed on the pure subphase (Figure 2C). Zhao and Feng^19^ have shown that PTX has a small surfactant effect since a compression isotherm of PTX applied directly at the air–water interface from the chloroform solution is measurable with a maximum surface pressure of 10 mN m^–1^. Thus, PTX can adsorb to some extent from the subphase at the interface at high areas per molecule especially in the gas phase but is expelled upon compression at surface pressures higher than ca. 10 mN m^–1^. Apparently, in the case of negatively charged DMPS monolayers, the interactions of PTX, which is an uncharged molecule and therefore electrostatic interactions are not taking place, are not as strong as for positively charged EPI.

For the mixture of PTX and EPI, the interactions are determined by the drug, which imposes a more pronounced effect on the monolayer of a given phospholipid. In the case of DPPC and cholesterol monolayers, the mixture of the drugs behaves more similarly to PTX, since for these two lipids, paclitaxel is affecting their surface properties more than epirubicin. The isotherms of a DPPC monolayer formed on the PTX and PTX + EPI subphase are practically the same in terms of their shape and position (Figure 2A). Compression modulus is slightly affected since its values are lower in the presence of the drug or drug mixture in the subphase, which suggests some changes in the organization of the DPPC molecules in the subphase. It is also confirmed by grazing incidence X-ray diffraction (GIXD) results collected for monolayers compressed to 30 mN m^–1^. DPPC monolayers on the pure water subphase exhibit typical two Bragg peaks pointing to the rectangular crystal lattice (Figure 3A).^78,79^ Although the presence of the mixture of the drugs in the subphase does not change the crystal lattice parameters, the intensity of the ⟨0,2⟩ peak is decreased and some parameters are also affected, e.g., the range of crystallinity in the ⟨0,2⟩ direction decreases quite significantly (Table 1). It proves that the drugs may decrease the extent of order in the condensed domains of DPPC.

**Figure 3:**
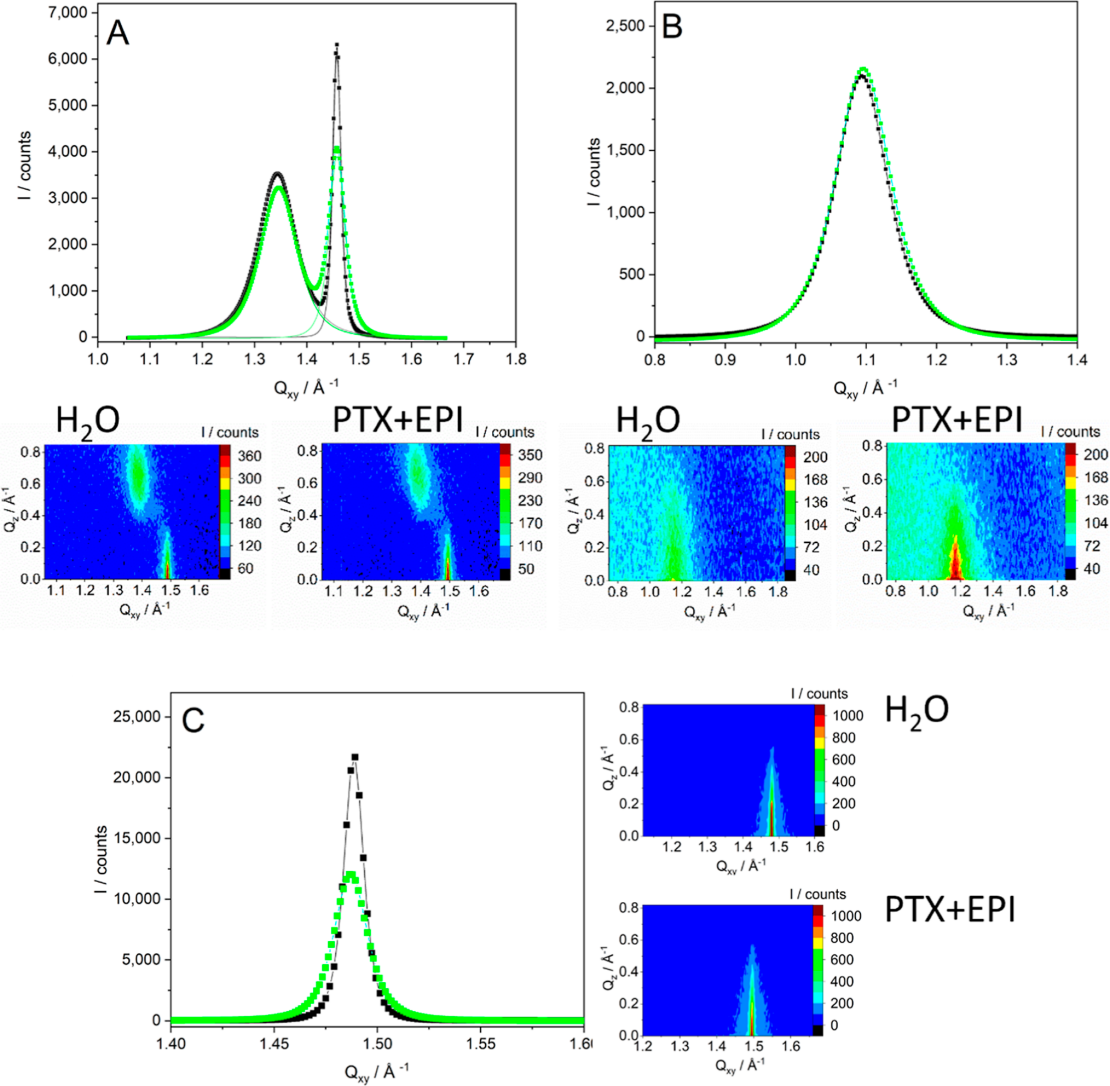
GIXD data: Bragg profiles for (A) DPPC, (B) cholesterol, and (C) DMPS monolayers on the pure water subphase (black) and the water subphase containing 10^–6^ mol/L PTX + EPI (green) collected at 30 mN m^–1^. The solid line is a Lorentz fit to the experimental data. *Q*_*z*_–*Q*_*xy*_ intensity maps are also included.

**Table 1:** Characteristic GIXD Parameters of DPPC, Cholesterol, and DMPS Langmuir Monolayers at 30 mN m^–1^ Formed on Subphases Containing 10^–6^ mol/L PTX + EPI; *Q*_*xy*_—Location of the Intensity Maximum of the Bragg Peak, *Q*_*z*_—Location of the Intensity Maximum of the Bragg Rod, *L*_*xy*_—the Range of 2D Crystallinity, *a*, *b*, and γ—Lattice Parameters, and *A*_uc_—Area of the Unit Cell

subphase	*Q*_*xy*_/Å^–1^	*Q*_*z*_/Å^–1^	*L*_*xy*_/Å	*d*/Å	*a*, *b* (Å)	γ (deg)	τ (deg)	*A*_uc_/Å^2^
DPPC								
water	⟨0,2⟩ 1.458	0	382 ± 9	4.31	5.57	90	31.6	48.98
	⟨1,1⟩ 1.344	0.693	82 ± 4	4.68	8.62			
10^–6^ mol/L PTX + EPI	⟨0,2⟩ 1.458	0	195 ± 9	4.31	5.55	90	31.0	47.86
	⟨1,1⟩ 1.346	0.681	85 ± 4	4.67	8.62			
Chol								
water	1.094	0	80 ± 1	5.74	6.63	120		38.10
10^–6^ mol/L PTX + EPI	1.096	0	76 ± 1	5.73	6.62	120		37.96
DMPS								
water	1.489	0	638 ± 9	4.22	4.87	120		20.57
10^–6^ mol/L PTX + EPI	1.487	0	370 ± 8	4.23	4.88	120		20.61

The effect of the drug mixture on cholesterol monolayers is also governed by PTX and is the most visible at the beginning of compression (Figure 2B). In the presence of the mixture of the drugs, the lift-off area is strongly shifted to the greater values, which is typical for the effect of PTX. At higher surface pressures, the drugs are removed from the monolayer and the isotherms converge, although the compression modulus decreases slightly but remains within the LC state region (Table S1). As a result, the crystal lattice parameters (Table 1) and the intensity of the typical, single Bragg peak for cholesterol^76,80–82^ remain unchanged (Figure 3B). Therefore, it may be concluded that the condensed domains of cholesterol at 30 mN m^–1^ are still present at the interface and contribute to the observed GIXD signal.

The situation is different for DMPS (Figure 2C), since EPI affects the DMPS monolayer more than PTX due to the presence of electrostatic interactions with the PS headgroup. However, in the presence of the mixture of the drugs, the lift-off area is strongly shifted to the greater values, which is typical for the effect of PTX. Further compression above 20 mN m^–1^ results in the isotherm shape exactly the same as the isotherm obtained in the presence of EPI alone. Additionally, the interactions of the two drugs applied simultaneously lead to a more liquid organization of a monolayer, as evidenced by the significantly decreased values of compression modulus (inset in Figure 2C and Table S1) and the lack of typical LC domains of DMPS at the surface pressure of approximately 10 mN m^–1^ visualized by Brewster angle microscopy (Figure S1).^7,83,84^ Instead, the domains start to appear at a much higher surface pressure of 29 mN m^–1^, proving the shift of the phase transition to higher surface pressures. The changes in the organization of the DMPS monolayer exposed to the mixture of the drugs observed in the mesoscale using BAM are also confirmed by GIXD results. DMPS molecules compressed to 30 mN m^–1^ on pure water organize themselves at the interface in the hexagonal lattice, as proved by a single Bragg peak centered at 1.489 Å^–1^ (Figure 3C and Table 1), which stays in excellent agreement with previous results.^9,85^ The presence of the mixture of the drugs in the subphase does not affect the peak position or unit cell, but the intensity of the peak decreases significantly. Lattice parameters are not affected, but the range of crystallinity decreases quite significantly (Table 1). On the π–*A* isotherm, the surface pressure, at which the GIXD measurement is performed (30 mN m^–1^), is just in the middle of the surface pressure plateau of the LE–LC transition that is shifted upward with respect to the value of surface pressure (Figure 2C). Exactly the same observations were made previously for doxorubicin, an enantiomer of EPI, interacting with DMPS monolayers.^9^ It suggests that the effect of EPI is dominating the interactions of the mixture of the two drugs with the DMPS membrane. It is also suggested by the results of the GIXD studies performed for DMPS monolayers exposed to single drugs (Figure S2 and Table S2). The GIXD signal and lattice parameters for the DMPS monolayers formed on the subphase containing PTX + EPI, EPI, or PTX alone are very similar, but the reduction in the GIXD signal is the same for EPI and PTX + EPI.

### 3.2 Three-Component Model Membranes at the Air–Water Interface

In order to provide more accurate models of breast cancer cell membranes, three-component monolayers at the air–water interface were prepared. The lipid molar composition of such model systems was designed in such a way that it reflects the most important phospholipid components of breast cancer cell membranes in terms of both the content of the polar heads of the phospholipids and the presence of cholesterol as a relevant component of the membranes.^28,29^ As a result, the model system was composed of DPPC:Chol:DMPS in a 4:4:2 molar ratio. Such a mixed membrane forms stable monolayers at the air–water interface with well-organized molecules forming the liquid-condensed phase (Figure 4A and Table S1). The isotherm of the ternary monolayer reflects the surface properties of the individual components (Figure S3). The excess area (*A* ^Exc^) values are negative at the beginning of compression (Figure 4B), which suggests more attractive interactions between the different components in the mixed monolayer compared to individual components.^51^ However, with the increasing surface pressure, the excess area values become slightly positive or close to 0, which points to less attractive interactions.

**Figure 4:**
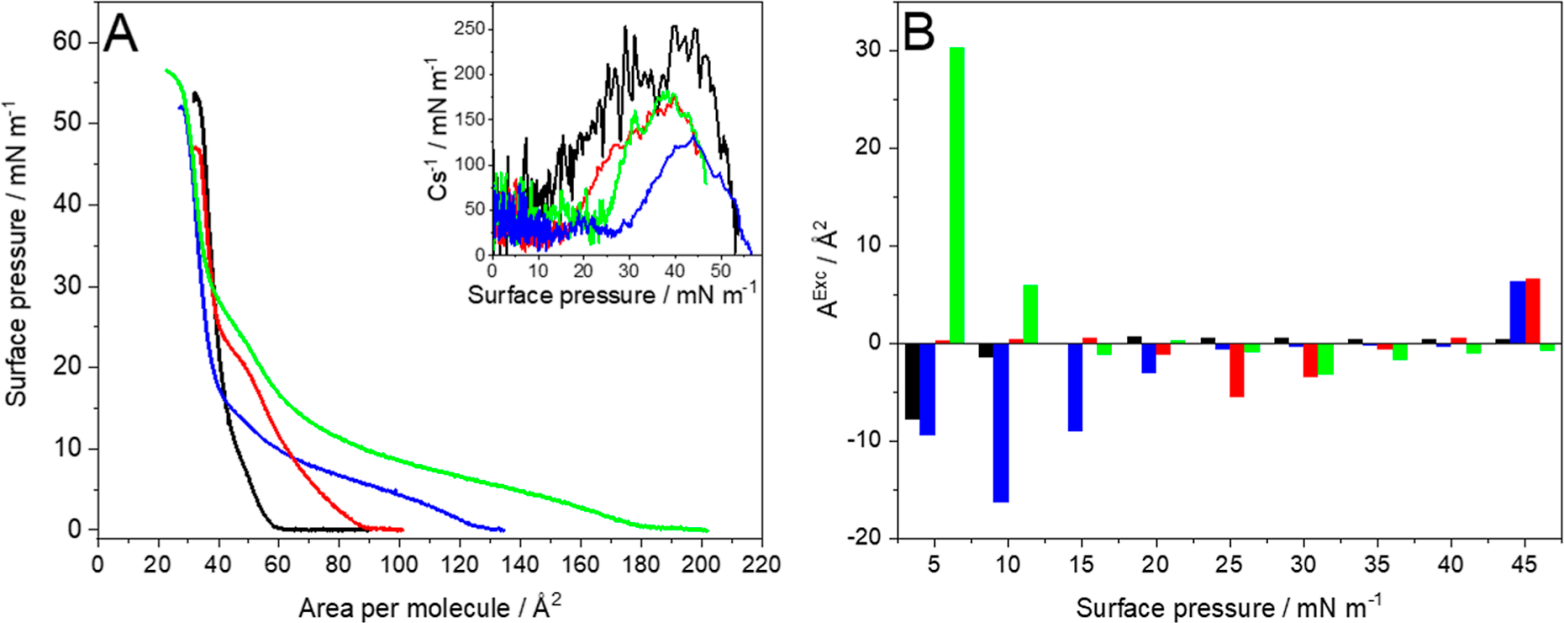
(A) Surface pressure–area per molecule (π–*A*) isotherms and (B) excess area (*A* ^Exc^) values calculated at selected surface pressure values for DPPC:Chol:DMPS 4:4:2 monolayers on the pure water subphase (black) and the water subphase containing 10^–6^ mol/L PTX (blue), 10^–6^ mol/L EPI (red), and 10^–6^ mol/L PTX + EPI (green). Inset in A: reciprocal of compression modulus vs surface pressure plot. (*T* = 21 ± 1 °C).

The ternary monolayers are strongly affected by both individual medicines and their mixtures (Figure 4). The presence of PTX in the subphase causes the shift of the lift-off area to the higher values of area per molecule, but again, at the surface pressure of approximately 15 mN m^–1^, the isotherm crosses and becomes similar to the isotherm of the ternary layer on the pure water subphase. In the case of EPI presence in the subphase, the lift-off area is not that significantly shifted as in the presence of PTX, but the shape of the isotherm is different. The LE phase appears in the isotherm with a *C*_s_^–1^ lower than 100 mN m^–1^ and a transition plateau occurs at a higher surface pressure of approximately 22 mN m^–1^. Again, the presence of EPI as for pure DMPS favors the liquid phase. At higher surface pressures, the isotherms converge. Additionally, the values of excess area become positive for the individual drugs present in the subphase at the highest surface pressures, which suggests more repulsive interactions between the different components in the mixed layer compared with single components. When the two medicines are present in the subphase, the effect is the strongest, and the observed effects reflect the changes in the shape of isotherms noted for the two individual anticancer agents. The lift-off is significantly shifted to a much larger area per molecule (Figure 4A). Additionally, in the initial stages of compression, the excess area in the presence of the two anticancer agents attains highly positive values, which implies the presence of much more repulsive interactions between the components in the mixed layer compared to those in single components (Figure 4B). As a result, the synergy defined by eq 6 is observed (Table 2). In the case of lower surface pressures, the difference in the area per molecule in the presence of the drug mixture (Δ*A*_PTX+EPI_) is much greater than the sum of the differences in the area per molecule calculated for the individual drugs (Δ*A*_PTX_ + Δ*A*_EPI_). It means that the presence of the two drugs in the subphase induces increased interactions with the model membranes at the air–water interface compared to the sum of the effects of the individual drugs. With the increasing compression and organization of the model membrane, this effect decreases and the observed synergy is smaller (Table 2). Similar observations were made by Bartkowiak et al., who followed the synergistic effect of simvastatin coadministered with doxorubicin on the models of cancerous cell membranes at the air–water interface.^52^

**Table 2:** Changes in the Area per Molecule at Selected Values of Surface Pressure for DPPC:Chol:DMPS 4:4:2 Monolayers on Subphases Containing 10^–6^ mol/L PTX, 10^–6^ mol/L EPI, and 10^–6^ mol/L PTX + EPI

area change	*A*_5mN/m_/Å^2^	*A*_10mN/m_/Å^2^	*A*_30mN/m_/Å^2^
Δ*A*_PTX_	45.2 ± 1.2	15.1 ± 0.9	–3.5 ± 0.9
Δ*A*_EPI_	22.0 ± 0.3	16.9 ± 0.1	–0.7 ± 1.1
Δ*A*_PTX_ + Δ*A*_EPI_	67.2 ± 1.5	32.0 ± 1.0	–4.2 ± 2.0
Δ*A*_PTX+EPI_	87.3 ± 0.4	42.7 ± 0.3	–0.5 ± 1.1

Further information about the influence of the two anticancer drugs with model breast cancer membranes was obtained by the consecutive compression–expansion cycles (Figure 5A). This type of experiments may provide suggestions on the possible formation of aggregates and the reversibility of the compression–expansion processes determined by the adhesion forces and the viscosity of the layers.^55,56^ The lack of hysteresis and the values of thermodynamic parameters close to 0 point to ideally behaving layers. It can be observed for ternary monolayers formed on the pure water subphase (Table 3). It should be noted that the value of the free energy of hysteresis (Δ*G* ^hys^) in the presence of both drugs is the most negative, which points to the presence of cohesive intra- and intermolecular forces within the components of the monolayer, probably due to the formation of some aggregates and/or assemblies. The negative value of *T*Δ*S* ^hys^ additionally proves the presence of entropically unfavorable interactions. It may suggest the formation of some complexes between the two drugs, which contributes to the irreversibility of the whole system. The synergistic interaction of PTX and EPI with the model membrane is enthalpically favorable, as shown by the negative values of (Δ*H* ^hys^).

**Figure 5:**
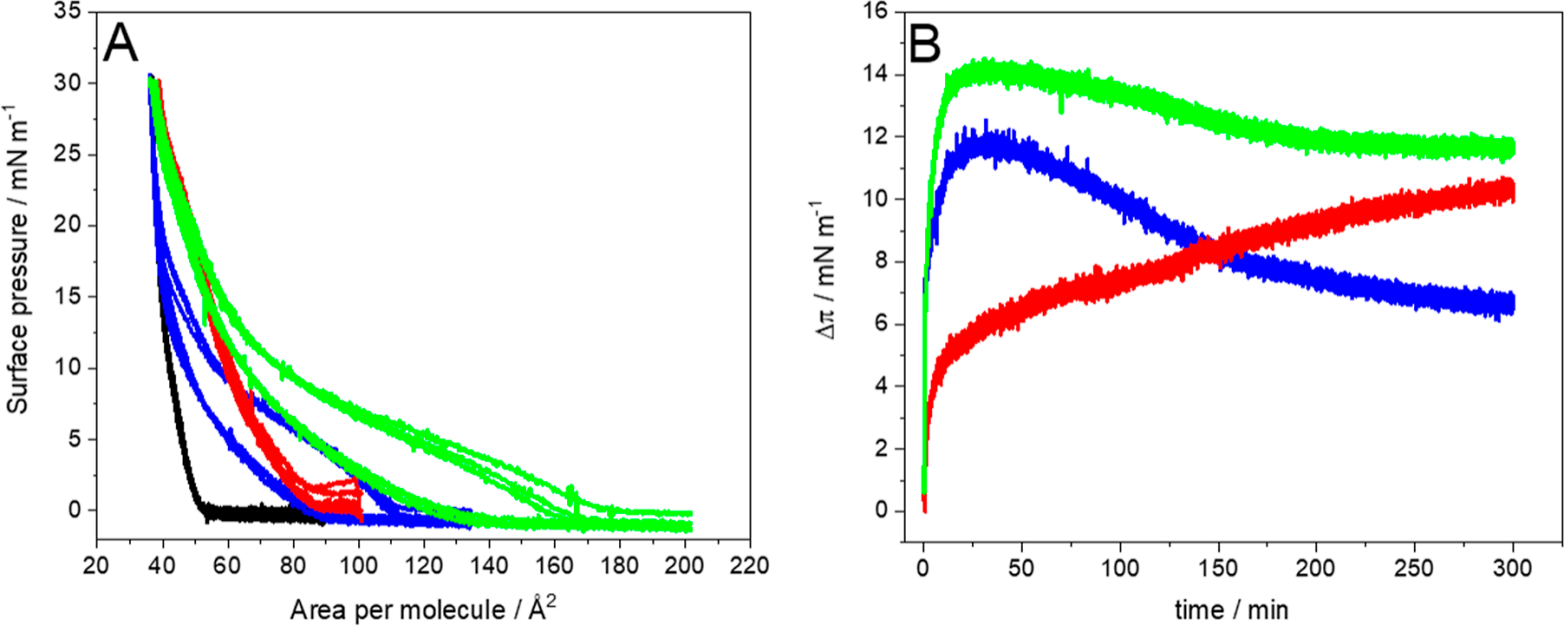
(A) Consecutive compression–expansion cycles; (B) the difference in surface pressure in time (Δπ) for DPPC:Chol:DMPS 4:4:2 Langmuir monolayers compressed to 30 mN m^–1^ on the water subphase after the addition of PTX (blue), EPI (red), and PTX + EPI (green) (*T* = 21 ± 1 °C). Δπ is calculated as the difference between the π value of the lipid monolayer in the presence of the drugs and the π value of the pure lipid monolayer.

**Table 3:** Thermodynamic Functions of Hysteresis Calculated between π = 1 mN m^–1^ and π = 30 mN m^–1^ for DPPC:Chol:DMPS 4:4:2 Langmuir Monolayers on the Pure Water Subphase and Subphases Containing 10^–6^ mol/L PTX, 10^–6^ mol/L EPI, and 10^–6^ mol/L PTX + EPI

subphase	Δ*G*_comp_/kcal·mol^–1^	Δ*G*_exp_/kcal·mol^–1^	Δ*G* ^hys^/kcal·mol^–1^	*T*Δ*S* ^hys^/kcal·mol^–1^	Δ*H* ^hys^/kcal·mol^–1^
water	0.22 ± 0.02	0.19 ± 0.01	–0.02 ± 0.00	–0.14 ± 0.00	–0.16 ± 0.00
10^–6^ mol/L PTX	0.95 ± 0.14	0.50 ± 0.06	–0.45 ± 0.09	–2.35 ± 0.20	–2.80 ± 0.29
10^–6^ mol/L EPI	0.78 ± 0.00	0.71 ± 0.03	–0.07 ± 0.03	–0.42 ± 0.16	–0.48 ± 0.19
10^–6^ mol/L PTX + EPI	1.77 ± 0.17	1.16 ± 0.14	–0.61 ± 0.03	–2.43 ± 0.05	–3.05 ± 0.08

The interactions of the two anticancer drugs applied individually and simultaneously with the mixed layer in the longer time scale were observed during the stability experiments (Figure 5B). In this type of experiment, the model membranes were first compressed to the surface pressure of 30 mN m^–1^, which assures that the molecular organization and elastic properties of monolayers at the air–water interface reflect the properties of the bilayers in biological membranes.^53,54^ Just after the injection of the drug/drug mixture, the surface pressure increased significantly for all the systems investigated (Figure 5B). Further changes of the surface pressure in time depended on the anticancer drug. In the presence of PTX, the surface pressure dropped in time and further stabilized at approximately Δπ = 6 mN m^–1^ after 3 h. On the contrary, the interactions with EPI led to the gradual, constant increase in surface pressure in time. The injection of PTX + EPI into the subphase resulted in the highest increase in the Δπ and was followed by only a very slight decrease in the surface pressure in time. However, in several hour time scales, the surface pressure value in the presence of PTX + EPI was higher than the values recorded in the presence of the individual drugs.

### 3.3 Three-Component Model Membranes on a Solid Support

The interactions between the drugs and the reconstituted membrane as well as the resulting changes in viscoelastic properties were also evaluated by a quartz crystal microbalance with dissipation monitoring (QCM-D). Figure 6 presents the QCM-D data, illustrating both the frequency shifts (Figure 6A–C) and energy dissipation (Figure 6D–F) across various overtones, with the lighter colors representing lower overtones and the darker colors representing higher ones. Once the lipid bilayer exhibited stability in H_2_O, each drug was introduced individually into the cell, followed by exposure of the membrane to their mixture. A significant frequency drop was observed upon drug introduction, indicating drug–membrane interaction. For PTX (Figure 6B,E), a rapid frequency decline was noted, signifying immediate interaction with the membrane. The signal stabilized temporarily for up to 60 min, after which a frequency increase was observed, likely due to mass loss from the prism. After approximately 90 min, the signal stabilized with a negative frequency value. The minor differences in overtones suggest a uniform interaction of the drug with the lipid bilayer. Dissipation exhibited a transient increase, correlating with enhanced membrane fluidity, but thereafter decreased steadily, reaching negative values after 120 min. The small overtone differences here also imply homogeneous membrane changes. The data indicate that PTX is capable of integrating into the membrane’s structure, leading to its stiffening. The observed mass increase after 60 min could be related to water expulsion from the lipid bilayer, as a result of the membrane stiffening induced by the incorporated PTX. The observed mass decrease after 60 min could be related to water expulsion from the lipid bilayer as a result of membrane stiffening induced by the incorporated PTX.

**Figure 6:**
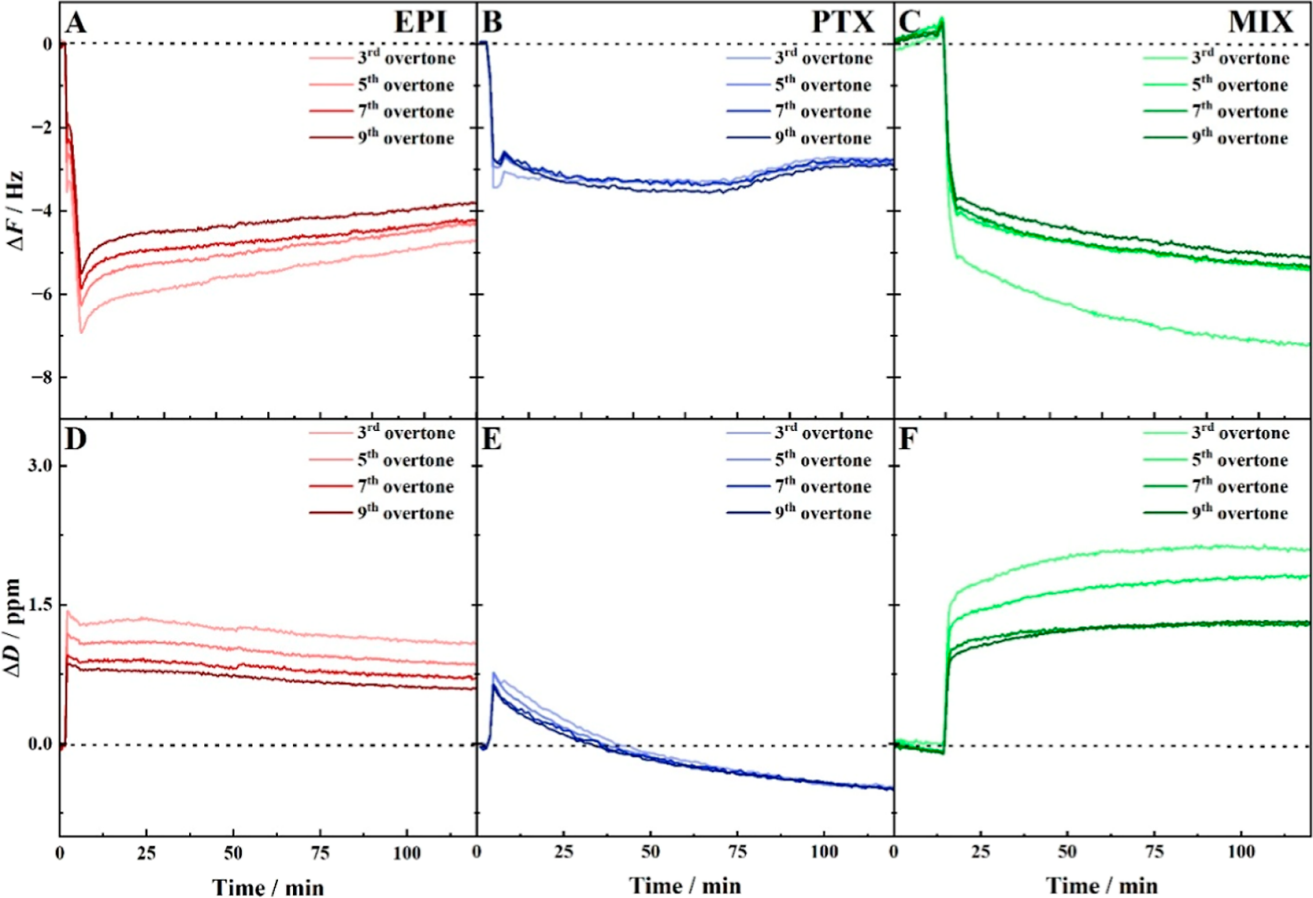
QCM-D lipid membrane covered sensor response to the presence of drugs (red curves: A,D—10^–6^ mol/L EPI; blue curves: B,E—10^–6^ mol/L PTX; green curves: C,F—10^–6^ mol/L PTX + EPI). Graphs A, B, and C show changes in the crystal frequency, while graphs D, E, and F show changes in energy dissipation over time. Measurements were performed in water. Overtones are indicated by variations in the intensity of the base color, from the darkest (highest overtone) to the lightest (lowest overtone).

The trends observed in the case of EPI (Figure 6A,D) present different characteristics compared to PTX. Initially, the frequency decrease associated with EPI is more pronounced, although the overall frequency change after 120 min is similar to the one observed for PTX (Figure 6B). However, there are significant differences across the overtones, with the most visible changes occurring at overtone 3. Each overtone oscillates at a different fundamental frequency, allowing it to provide information about the spatial distribution of mass changes associated with the sensor surface. For example, the third overtone is more sensitive to changes further from the surface, whereas the 11^th^ overtone is more sensitive to changes closer to it.^86^ This suggests that EPI may interact more strongly with the membrane surface, potentially indicating preferential binding with polar parts of the lipids. Regarding dissipation, there is minimal variation over time following the initial change. The observed increase in dissipation suggests an enhancement in structural disorder relative to the native membrane situation.

Significantly greater changes are observed for the drug mixture (Figure 6C,F). Both frequency and energy dissipation signals exhibit considerable changes immediately following the introduction of the drug mixture. The total frequency decrease, which exceeds the cumulative effect of the individual drugs, was noted for the third overtone. For the other overtones, the effect is smaller, yet the frequency shift remains lower than that observed for each drug individually. Nevertheless, the overall behavior and continuous decrease in the frequency may suggest a more efficient incorporation of both anticancer drugs into the membrane. It should be emphasized that this cumulative effect may be partially masked by the distinct dissipation behavior observed for the individual drugs. Changes in membrane stiffness are associated with different hydration states of the membrane and are directly related to the amount of water bound to it. The marked increase in dissipation indicates a pronounced enhancement of the membrane fluidity. Additionally, deviations across individual overtones are noticeable, particularly at overtone 3, suggesting that the interaction of the drug mixture with the membrane is heterogeneous. These observations imply that the combined drugs exert a significantly greater impact on the structural integrity of the lipid bilayer compared with the effects of the individual drugs.

The infrared spectra obtained via attenuated total reflectance spectroscopy (ATR-IR) were subsequently analyzed for the lipid membrane formed on a silicon substrate. This technique enabled the calculation of the average tilt angle of hydrocarbon chains of lipid molecules relative to the surface normal and provided insights into the specific membrane regions most affected by the drug or drug mixture.

Figure 7 displays the IR spectra of the membrane both before and 2 h after drug introduction. Three characteristic bands corresponding to the hydrophobic region of the membrane—specifically the lipid chains—were observed at 2955, 2920, and 2850 cm^–1^. These bands indicate that the membrane predominantly exists in an *all-trans* conformation, characteristic of a gel state.^87,88^ Notably, the positions of these bands did not undergo any significant shifts over time and upon drug exposure. However, by utilizing polarized light, the tilt angle of the hydrocarbon chains of lipid molecules relative to the surface normal was determined (Formulas S1–S5, Supporting Information, Figure S3).^89–91^ The calculated angle was 31 ± 1°, which decreased slightly to 28 ± 1° following the two hour exposition to PTX. This reduction in the tilt angle of hydrocarbon chains corresponds to an increase in membrane rigidity, as reflected in the decreased energy dissipation observed in the QCM data. Subsequently, the drug’s effect on the polar region of the membrane was examined (Figure 7). The spectrum for the model membrane (black) displayed two characteristic bands: one at 1733 cm^–1^, corresponding to the C=O stretching vibration, and another at 1618 cm^–1^, corresponding to the asymmetric stretching vibration of COO^–^ present in the serine moiety of the DMPS component.^92^ Upon drug addition, both bands exhibited a significant shift toward higher wavenumbers, indicating a decrease in membrane hydration. This effect was particularly pronounced for the COO^–^ band in serine, where a substantial shift was observed. These results suggest that in addition to the stiffening of the membrane, PTX also contributes to the removal of water molecules, thereby decreasing membrane hydration. This reduction in hydration was corroborated by QCM data, where a slight increase in frequency was observed approximately 60 min after drug introduction, suggesting a decrease in mass on the crystal, likely due to water expulsion. Therefore, it can be concluded that this drug integrates into the hydrophobic region of the membrane, leading to membrane stiffening in a manner analogous to that of cholesterol. In Figure S5 provided in the Supporting Information, the FTIR spectra of epirubicin and paclitaxel are shown. In both cases, some of the bands in the drug spectra overlap with those of the lipid membrane components. However, the drug concentration during the measurements was relatively low compared with the lipid concentration. Consequently, obtaining direct signals from the drug in the membrane spectrum would be very difficult, and therefore, it has a negligible impact on the positions of the lipid bands.

**Figure 7:**
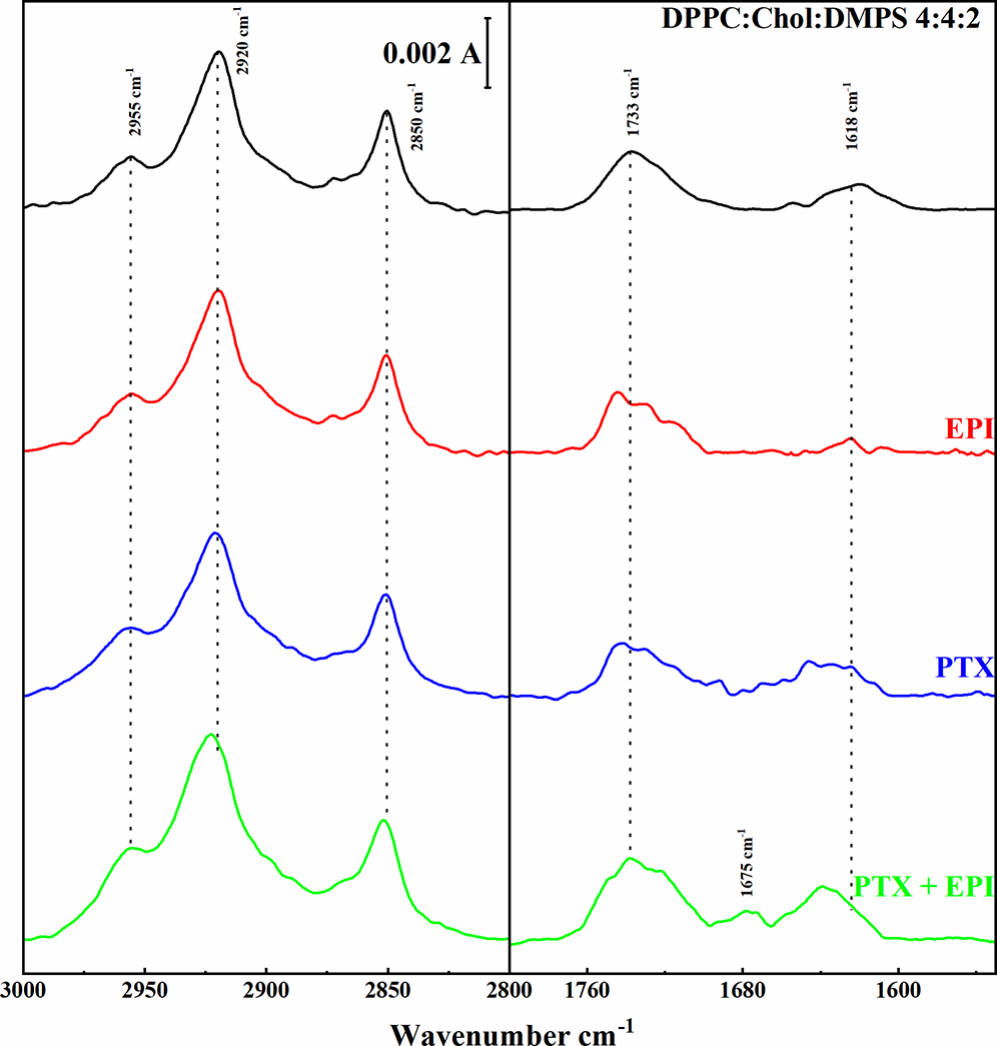
ATR spectra of model breast cancer membranes (black) showing the effect of 10^–6^ mol/L EPI (red), 10^–6^ mol/L PTX (blue), and 10^–6^ mol/L PTX + EPI (green) in the CH stretching region (left part of the figure) and polar headgroup region (right part of the figure). Experiments were performed in D_2_O on the hemisphere silicon prism.

A similar analysis was performed for EPI (Figure 7). No significant spectral changes were observed within the hydrophobic region, and the bands corresponding to the membrane before and after drug interaction are nearly identical. However, the analysis of the tilt angle of lipid molecules relative to the surface normal revealed notable changes. After EPI addition, the angle increased to 42 ± 3° (Figure S3), indicating that the membrane becomes less densely packed and exhibits increased fluidity—an observation corroborated by the quartz crystal microbalance data (the increased dissipation was noticed). In the polar region (Figure 7), the C=O band undergoes a shift similar to what was observed for PTX. Notably, the serine band decreases significantly and splits into two separate peaks at 1625 and 1609 cm^–1^. This suggests that EPI, which possesses both carboxyl and amino functional groups, may specifically interact with serine residues of DMPS. As a result, EPI may disrupt the distribution of water molecules deeper within the membrane, thereby reducing the hydration of the C=O band (observed at 1747 cm^–1^).^93^ This interpretation aligns with the QCM data, where the most pronounced changes in mass are observed at overtone 3, suggesting significant drug-induced alterations in the membrane structure. Therefore, it might be concluded that EPI, unlike PTX, prefers to be located in the hydrophilic part of the membrane.

The final stage of the study involved analyzing the impact of the drug mixture on the DPPC:Chol:DMPS 4:4:2 membrane. The IR spectrum in the CH band region reveals a pronounced effect of the drugs on the membrane structure (Figure 7). All characteristic bands shifted toward higher wavenumbers, indicating a transition of the membrane to a more fluid state, consistent with the *gauche* conformation typical of a liquid phase.^87,88,94^ Additionally, an increase in band intensity was observed, suggesting a significant increase in the tilt angle of the lipid molecules relative to the surface normal. Given that the dipole moment of the CH bands is oriented perpendicular to the lipid molecular axis, an increase in the overall membrane tilt angle causes the dipole moments of the given vibration to be more parallel to the surface normal, which causes an increase in the intensity of the bands. The average tilt angle of the lipid molecules in this case is approximately 58° ± 2° (Figure S3). However, it is important to note that the calculated angle exceeds the so-called magic angle (∼55°). Consequently, in this case, the membrane exhibits a completely random orientation and undergoes partial degradation. It is also possible that various types of conjugates, such as micellar systems, form on the surface.^95^ This substantial change in the tilt suggests that the membrane’s structural integrity is significantly disturbed, leading to a highly disordered, liquid-like state. In the spectral range from 1800 to 1550 cm^–1^ (Figure 7), the COO^–^ serine band exhibits behavior similar to that observed for EPI, indicating that EPI retains its interaction characteristics, even within the drug mixture. However, a new band appears at 1676 cm^–1^, likely corresponding to the formation of complexes due to the presence of both drugs in the solution. However, it is difficult to clearly determine the origin of this band. Interestingly, it is observed only when the two drugs are present. Due to the fact that the drugs-to-lipid ratio is relatively low, making it unlikely that a band arising directly from drug–drug aggregates would be detectable, it may be postulated that this band most likely originates from a drug–drug–lipid conjugate. In contrast, the C=O band displays a different behavior compared to the previous spectra. Alongside the band components characteristic of nonhydrated C=O, a component at 1722 cm^–1^ occurs, suggesting an increase in hydrogen bonding within this group. This phenomenon can be attributed to the increased tilt angle of the membrane and the resulting reduction in the molecular packing density. Consequently, a part of the lipid molecules becomes more exposed to the aqueous environment, leading to enhanced hydration of the C=O group. These observations are consistent with the calculated membrane tilt angle. These results clearly demonstrate that the drug mixture exerts a significantly more disruptive effect on the membrane compared to the effects observed when each drug is applied individually and might lead to a total disruption of the membrane.

### 3.4 Molecular Dynamics

In order to complement the experimental results and assess the drug aggregation and interaction with lipid monolayer components, the all-atom detail molecular dynamics (MD) method was employed. MD simulations enable us to determine the radial distribution functions between drug molecules and lipids, the average number of hydrogen bonds, and the order parameter in systems with varying configuration. The four distinct systems were investigated: pure membrane, two consisting of single drugs (PTX or EPI), and one containing their mixture. The lipid monolayer composition was the same in all systems and matches the experimentally studied one, i.e., DPPC:Chol:DMPS with a molar ratio 4:4:2. This set of simulations allowed for a comprehensive analysis of how the individual and combined properties of these lipids influence the interactions and behavior of drug molecules within the monolayer. The area per lipid (APL) in the simulations was set to 65 Å^2^ to reflect such a state of monolayer compression, in which the effect of the individual drugs and their mixture is the most significant and the drugs are still within the monolayer before they are expulsed at higher surface pressures (Figure 4A).

The radial distribution function (RDF) was calculated between the drug molecules (Figure 8D). The highest value of the *g*(*r*) was observed for pure EPI in the monolayer, which suggests the presence of aggregates. This is also visible in the snapshots from the simulations (Figure 8A). Interestingly, the tendency to form aggregates by anthracyclines has been previously suggested in the literature and it was attributed to the hydrogen bonding.^16,17,96^ Therefore, the average number of hydrogen bonds between drug molecules (Table 4) and other monolayer components (Tables S3–S5) was determined.

**Figure 8:**
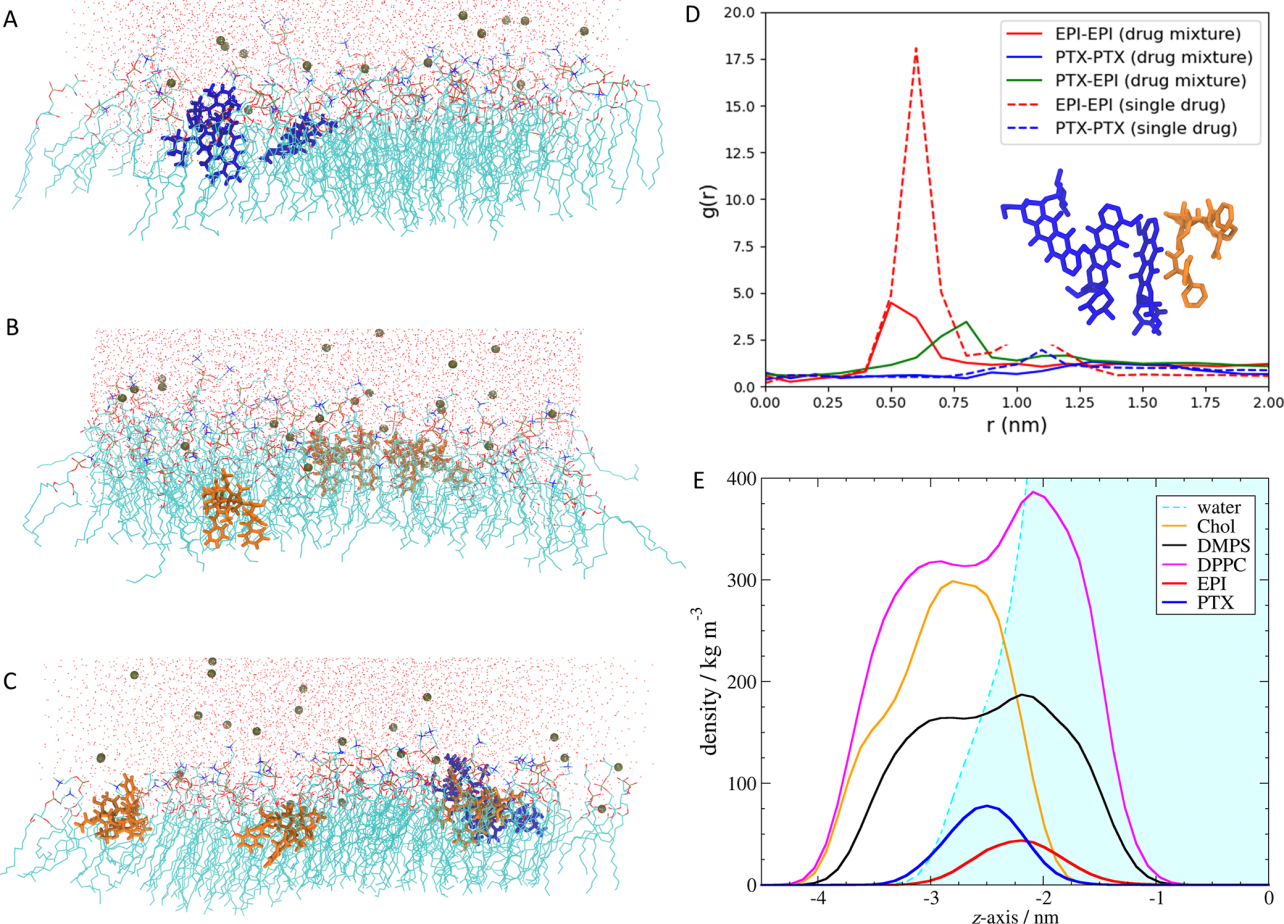
Snapshots showing the final configuration of the lipid monolayer consisting of (A) EPI (blue), (B) PTX (orange), and (C) mixture of EPI and PTX; (D) 2D radial distribution function in the *xy* plane between the drug molecules center of mass, calculated from MD simulations. The zoom of the EPI-PTX aggregate is shown in the inset. (E) The density distribution profile across the *z*-axis of the system containing the drug mixture.

**Table 4:** Average Number of H-Bonds between Drug Molecules

system	molecule pair	number of H-bond per molecule
single drug	PTX–PTX	0.32 ± 0.03
single drug	EPI–EPI	2.71 ± 0.04
drug mixture	PTX–PTX	0.31 ± 0.02
	EPI–EPI	2.58 ± 0.04
	PTX–EPI	0.20 ± 0.02

The average number of H-bonds (per molecule) between drug molecules in single drug systems was equal to 2.71 ± 0.04 and 0.32 ± 0.03 for EPI and PTX, respectively. This demonstrates that the aggregation of EPI molecules, observed in RDF results (Figure 8), is indeed hydrogen bond-mediated. The amount of hydrogen bonds between EPI molecules in the PTX + EPI system slightly decreased, reaching a value of 2.58 ± 0.04. This can result from aggregation between EPI and PTX molecules (Figure 8C). The number of H-bonds between PTX and EPI was relatively small, 0.20 ± 0.02. Therefore, it is evident that the PTX–EPI aggregation is not driven via hydrogen bonding interactions. Further investigation of the interaction between these two drugs reveals that 65% of the interparticle energy can be attributed to van der Waals interactions, while the remaining 35% is due to Coulomb potential. Additionally, on the basis of Tables S4 and S5, one can elucidate that all the lipids can interact with the drugs via H-bonds, which can stabilize their accumulation and aggregation in the monolayer. These results of MD simulations showing the formation of PTX–EPI aggregates (Figure 8C) are consistent with the results of experimental studies, especially ATR results obtained for supported ternary lipid membranes exposed to the PTX + EPI mixture, where a new band at 1676 cm^–1^ attributed to the formation of complexes/aggregates due to the presence of both drugs in the solution was observed (Figure 7). Also, the relative position of drugs within the lipid monolayer, suggested by the ATR results, was confirmed via density profiles determined from MD simulations (Figure 8E). The peak corresponding to EPI mass distribution is significantly shifted toward the water environment, i.e., EPI prefers the hydrophilic part of the monolayer. The position of the peak maximum coincides well with the maximum corresponding to the position of the DMPS and DPPC headgroups. In contrast, the PTX molecules occupy the hydrophobic region.

To obtain more insight into drug–lipid interactions, the RDF between them was calculated (Figure 9). The results show that both EPI and PTX dominantly accumulate around the DMPS molecules. In the drug mixture, however, the PTX molecule exhibits similar affinity for both DMPS and DPPC, while cholesterol has the lowest affinity for both drugs. These observations are consistent with the results of Langmuir studies of single component monolayers (Figure 2). The most significant effect is observed for DMPS monolayers exposed to EPI, although PTX also leads to a shift of the DMPS isotherm at the initial stages of compression and affects DPPC monolayers.

**Figure 9:**
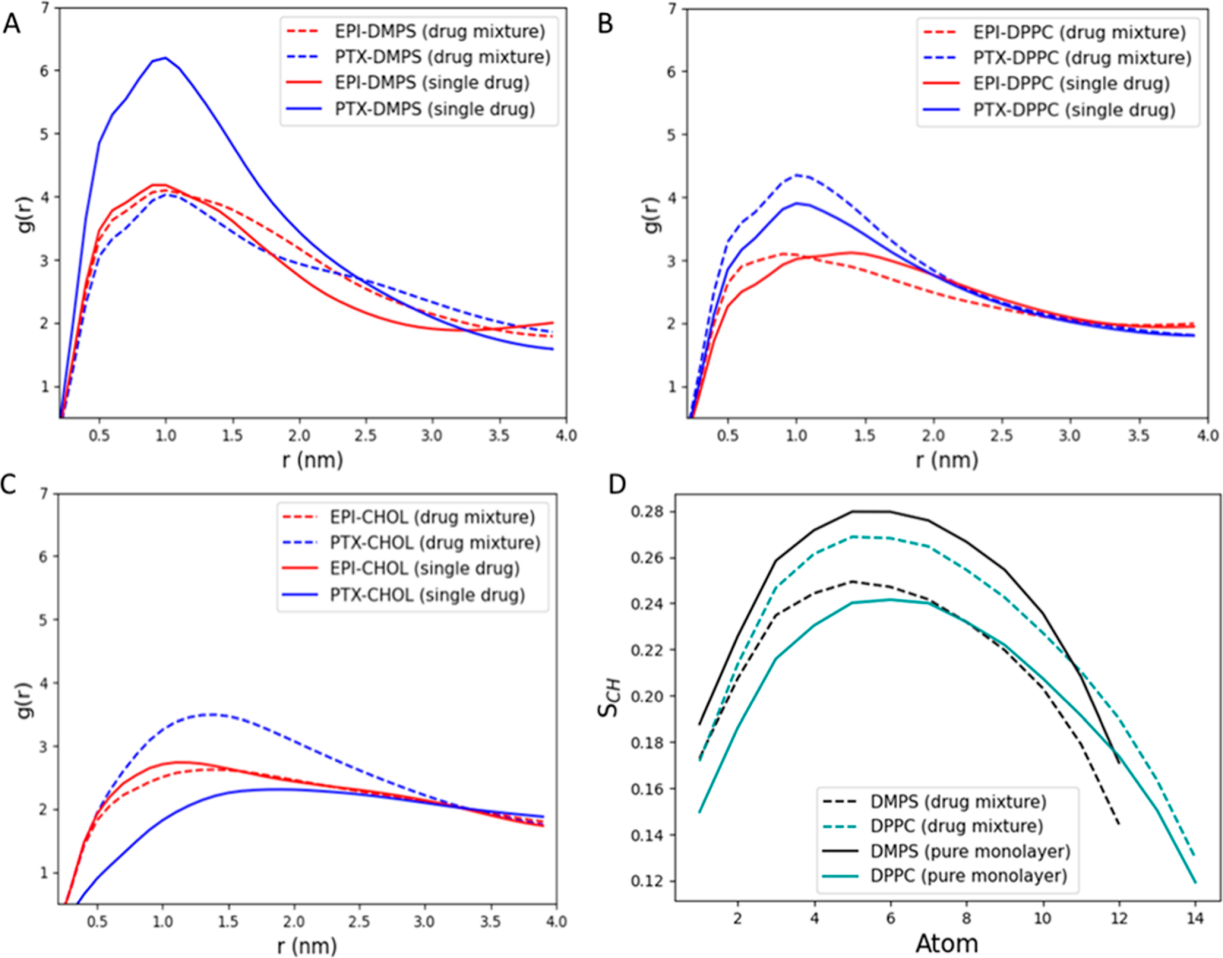
Radial distribution function between a drug molecule (EPI—red or PTX—blue) and a selected monolayer component: (A) DMPS, (B) DPPC, and (C) Chol; (D) the acyl chain order parameter, *S*_CH_, for the DMPS and DPPC calculated for the pure monolayer and monolayer containing the drug mixture. The *S*_CH_ parameters for single drug systems and RDFs for other monolayer components are in the Supporting Information (Figures S4 and S5).

In addition, we investigated the lipid distribution in a pure monolayer, as well as the effect of drug presence on lipid partitioning. We determined the RDFs for all the monolayer components and gather them in Figure S5. The clustering of DMPS lipids, observed in a pure monolayer, decreases upon incorporation of drug molecules. The clustering of DPPC lipids, as well as the DMPS–DPPC pair, is rather negligible. However, while Chol molecules have no clear preference either for DMPS or DPPC lipid, we observed a strong and long-ranged ordering between Chol itself.

The order parameter obtained from molecular dynamics simulations reflects the degree of ordering of the lipid chains in the membrane. The results of order parameter (Figure 9D) showed that the presence of drugs affects the lipid ordering, decreasing the overall *S*_CD_, in comparison to the pure membrane. Thus, the presence of the drug mixture significantly favors the liquid organization of DMPS. Lower-order parameters imply increased disorder and thus greater fluidity, in line with experimental results provided by surface pressure isotherms with the stabilized LE phase up to higher pressures, according to Brewster angle microscopy (Figure S1) as well as the observed decrease in the GIXD signal due to the shift of the LE–LC surface pressure plateau (Figure 3C). Increased fluidity can also be directly linked with the decreased values of compression modulus (Figure 2C and Table S1). On the other hand, MD results suggest increased ordering in case of DPPC. As the RDF results strongly suggest that drugs are mainly accumulated around DMPS, these observations might result from lipid partitioning and formation of DPPC rafts.

## 4 Conclusions

In this study, we have provided more detailed insight into the molecular-level understanding of the synergistic action of paclitaxel (PTX) and epirubicin (EPI) on biomimetic breast cancer cell membranes. These effects were elucidated through a combination of advanced interfacial characterization techniques including Langmuir monolayer studies at the air–water interface complimented with GIXD and BAM imaging as well as QCM, and ATR spectroscopy for supported bilayers, additionally proved by molecular dynamics simulations. Our findings clearly demonstrate that the combination of PTX and EPI compared to the effect of individual drugs significantly alters the physicochemical properties of phospholipid monolayers at the air–water interface, leading to the increased membrane fluidization and promoting irreversible aggregate formation. It has been shown that while neutral PTX interacts with all membrane components, positively charged EPI exhibits significant interactions predominantly with a negatively charged DMPS lipid. However, the observed synergy is mostly driven by drug clustering within the lipid bilayer, formation of irreversible aggregates, and the decreased ordering of DMPS. As proved by MD simulation, apart from electrostatic interactions, the formation of hydrogen bonding between the drug molecules is crucial for the formation of the aggregates leading to the observed synergy in the model system.

These insights are crucial for the rational design of novel drug delivery systems that can exploit or modulate drug–membrane interactions to enhance therapeutic efficacy. For instance, understanding the mechanisms of membrane fluidization and aggregation induced by this drug combination could inform the development of stimuli-responsive nanocarriers designed to destabilize cancer cell membranes upon drug release. Future research could focus on translating these findings to more complex *in vitro* and *in vivo* models as well as exploring the design of materials with tailored surface properties to optimize the delivery of synergistic drug combinations to cancer cells.

## Supplementary Material



## Notes

Formulas for the calculation of tilt angles of phospholipids in supported bilayers based on ATR spectra; characteristic parameters of phospholipid Langmuir monolayers formed on subphases containing PTX, EPI, and their mixture; BAM images of DMPS monolayers; GIXD data and parameters for DMPS monolayers; ATR spectra in the CH region for the DPPC:DMPS:Chol 4:4:2 membrane; and *S*_CH_ parameters for single drug systems determined from MD simulations (PDF)
