# Supplementary material for: Synergistic Effect of Paclitaxel and Epirubicin Coadministration—Insight into the Mechanisms of Interactions with Model Breast Cancer Cell Membranes
Source: Langmuir. 2025 Sep 29;41(40):27106–22. doi: 10.1021/acs.langmuir.5c02558 (PMC12529955; doi:10.1021/acs.langmuir.5c02558)
Supplement: Supplementary file 1 [file la5c02558_si_001.pdf]

Synergistic effect of paclitaxel and epirubicin co-administration – insight into the mechanisms of interactions with model breast cancer cell membranes.

Damian Dziubak[†1], Paulina Kaczmarczyk[†1], Izabella Leszczyńska[2], Piotr Batys[2], Philippe Fontaine[3], Dorota Matyszewska[1]*

[1]*University of Warsaw, Faculty of Chemistry, Biological and Chemical Research Centre, Żwirki i Wigury 101, 02089 Warsaw, Poland*

[2]*Jerzy Haber Institute of Catalysis and Surface Chemistry, Polish Academy of Sciences, Niezapominajek 8, 30239 Krakow, Poland*

[3]*Synchrotron Soleil, L'Orme des Merisiers, Départementale 128, 91190 Saint-Aubin, France*

The average tilt angles of the acyl chain and the surface normal were calculated based on the intensity of the $\upsilon_{as}(CH_2)$ band using IR polarization (*p* and *s*). First, the amplitudes of spatial components of the electric field (E_x, E_y, E_z) were calculated using these equations:

$$E_x = \frac{2\cos\theta\sqrt{sin^2\theta - n_{3/1}^2}}{\sqrt{1-n_{3/1}^2}\sqrt{\left(1+n_{3/1}^2\right)sin^2(\theta) - n_{3/1}^2}} \tag{1}$$

$$E_y = \frac{2\cos\theta}{\sqrt{1-n_{3/1}^2}} \tag{2}$$

$$E_z = \frac{2n_{3/2}^2\sin\theta\cos\theta}{\sqrt{1-n_{3/1}^2}\sqrt{\left(1+n_{3/1}^2\right)sin^2(\theta) - n_{3/1}^2}} \tag{3}$$

Where:

E_x, E_y, E_z – components of the electric field;

θ – angle of incidence of IR beam at the solid–liquid interface;

n – refractive indices of the internal reflection element (1 – Si prism, 2 – lipid membrane, 3 – aqueous solution).

Knowing the values of the individual components of the electric field, it was possible to calculate the parameters:

$$S_{chain} = \frac{2(E_x^2 - RE_y^2 + E_z^2)}{(3\,cos^2\alpha - 1)(E_x^2 - RE_y^2 - 2E_z^2)} \quad (4)$$

$$\theta_{chain} = \cos^{-1}\sqrt{\frac{2S_{chain} + 1}{3}} \quad (5)$$

Where:

S_{chain} – order parameter of lipids' hydrocarbon chain;

α - the transition dipole moments of $\upsilon_{as}(CH_2)$ to the molecular axis (in this case 90°);

R – dichroic ratio defined as absorbance of p-polarized and s-polarized light of the $\upsilon_{as}(CH_2)$ band;

θ_{chain} - average tilt angle of the acyl chains.

Table S1 Characteristic parameters of phospholipid Langmuir monolayers formed on subphases containing PTX, EPI and their mixture.

**Subphase**	A_0 / Å^2	$A_{5mN/m}$ / Å^2	$A_{30\,mN/m}$ / Å^2	A_{coll} / Å^2	π_{coll} / mN m^{-1}	Cs_{max}^{-1} / mN m^{-1}
			**DPPC**			
water	51.0±1.6	75.5±1.3	40.9±0.2	25.1±0.0	60.4±1.3	183±4
10^{-6} mol/L PTX	47.3±3.8	144.3±1.9	36.9±1.1	24.2±0.2	63.9±0.7	135±18
10^{-6} mol/L EPI	52.2±2.7	80.9±1.3	40.2±0.5	21.8±0.5	62,1±1.6	180±12
10^{-6} mol/L PTX+EPI	50.8±3.7	127.2±4.5	39.9±1.5	24.9±0.3	62.9±1.2	129±1
			**Chol**			
water	40.5±0.3	39.8±0.2	36.7±0.1	35.2±0.1	45.7±1.6	426±16
10^{-6} mol/L PTX	42.1±1.4	45.7±0.2	36.2±0.4	34.7±0.2	42.8±1.3	384±21
10^{-6} mol/L EPI	42.3±0.6	42.0±0.0	37.6±0.2	35.8±0.5	43.4±0.7	298±17
10^{-6} mol/L PTX+EPI	43.4±1.1	56.8±2.3	35.7±0.1	32.9±0.6	44.2±1.0	262±28
			**DMPS**			
water	37.0±0.3	62.4±0.2	32.1±0.8	23.7±0.5	59.9±3.8	291±27
10^{-6} mol/L PTX	35.3±1.6	140.2±0.0	30.1±0.9	21.9±0.1	64.0±0.4	183±9

10^{-6} mol/L EPI	39.0±1.1	117.7±1.6	53.2±1.2	18.9±1.6	63.0±0.4	126±18
10^{-6} mol/L PTX+EPI	40.2±1.4	162.5±0.5	52.0±0.1	13.6±1.1	62.0±0.2	108±9
**DPPC:Chol:DMPS 4:4:2**						
water	45.3±0.1	51.2±0.2	38.1±0.3	32.6±0.8	51.7±3.0	248±0
10^{-6} mol/L PTX	42.8±0.0	95.1±0.0	34.7±0.4	28.7±0.5	52.7±1.2	177±1
10^{-6} mol/L EPI	44.5±1.3	71.9±0.9	37.5±0.5	33.5±0.2	46.2±0.9	182±4
10^{-6} mol/L PTX+EPI	45.4±2.1	137.2±0.9	37.7±0.4	25.9±0.8	55.8±0.9	132±16

The morphology of the DMPS monolayers on pure water subphase or subphase with the PTX+EPI 10^{-6} mol/L solution was observed by Brewster angle microscopy (BAM) (Fig. S1). The Nanofilm Ep3 equipment with an UltraBAM objective (Accurion, Germany) was employed to record the images simultaneously with the compression of the layers at the air-water interface. Each image represents a field-of-view of 800 μm x 430 μm and was captured with the lateral resolution of 2 μm.

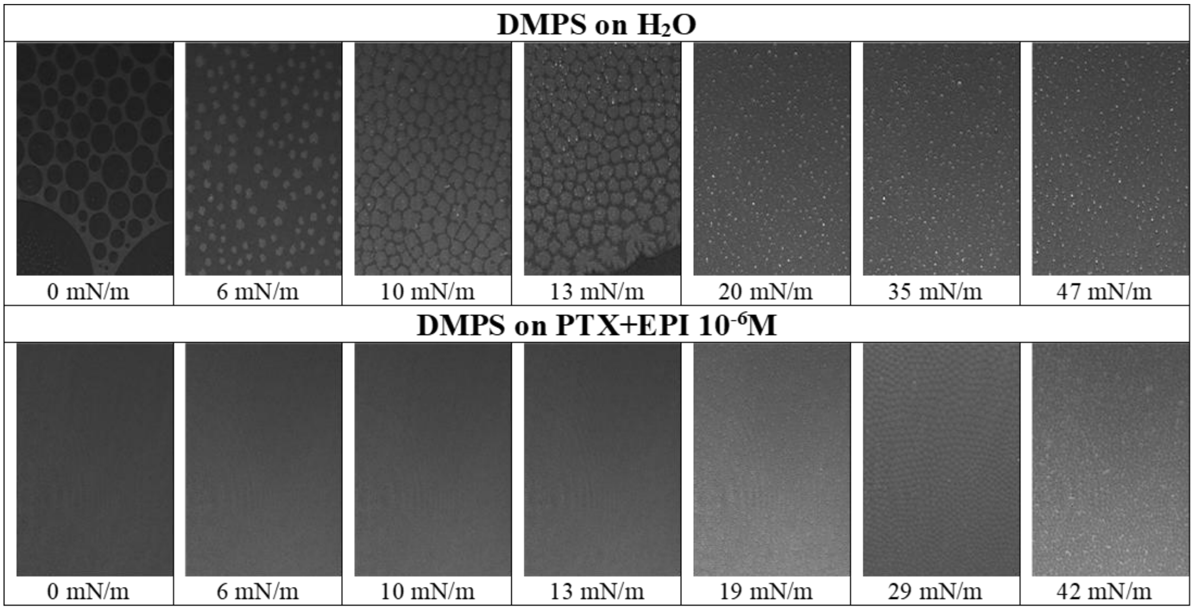

Figure S1 BAM images of DMPS monolayers formed on pure water subphase (upper row) and subphase containing 10^{-6} mol/L PTX+EPI (lower row).

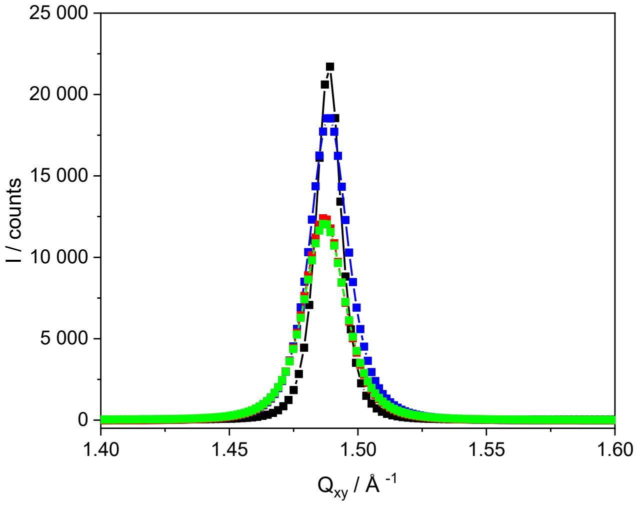

Figure S2 GIXD data Bragg profiles for DMPS monolayers on pure water subphase (black) and water subphase containing 10^{-6} mol/L PTX (blue), 10^{-6} mol/L EPI (red), 10^{-6} mol/L PTX+EPI (green). The solid line is a Lorentz fit to the experimental data.

Table S2 Characteristic GIXD parameters of DMPS Langmuir monolayers formed on subphases containing 10^{-6} mol/L PTX, 10^{-6} mol/L EPI, and 10^{-6} mol/L PTX+EPI; Q_{xy} – location of the intensity maximum of the Bragg peak, Q_z – location of the intensity maximum of the Bragg rod, L_{xy} – the range of 2D crystallinity, a, b, γ – lattice parameters, A_{uc} – area of the unit cell (per one hydrocarbon chain).

Subphase	Q_{xy} /Å^{-1}	Q_z /Å^{-1}	L_{xy} /Å	d / Å	a, b (Å)	γ (°)	A_{uc} /Å^2
water	1.489	0	638±9	4.22	4.87	120	20.57
10^{-6} mol/L PTX	1.489	0	407±8	4.22	4.87	120	20.57
10^{-6} mol/L EPI	1.487	0	376±8	4.23	4.88	120	20.62
10^{-6} mol/L PTX+EPI	1.487	0	370±8	4.23	4.88	120	20.61

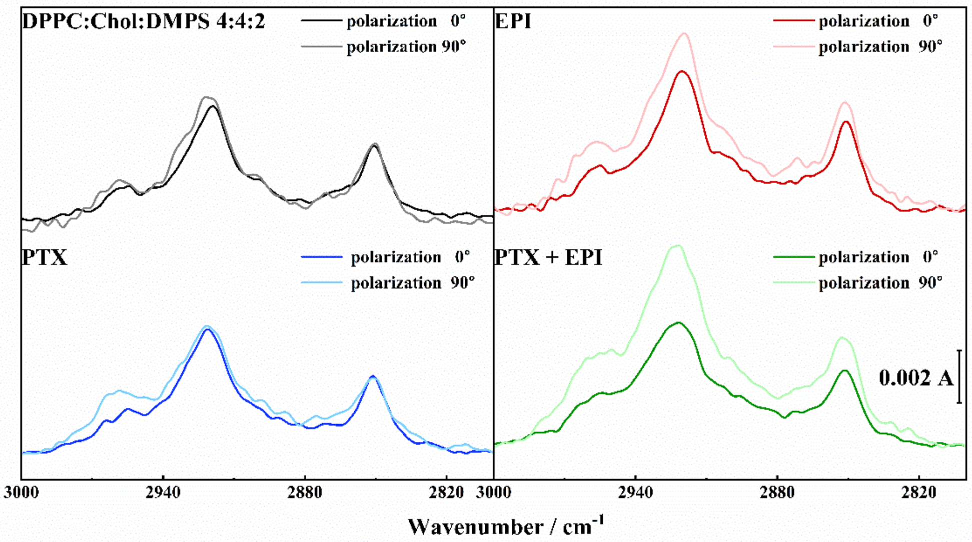

Figure S3. ATR spectra in the CH region, recorded for the DPPC:DMPS:Chol 4:4:2 membrane and for the membrane after 2 hours of exposure to 10^{-6} mol/L EPI, 10^{-6} mol/L PTX, or 10^{-6} mol/L PTX+EPI as a mixture of drugs (MIX). The experiments were performed using a silicon hemisphere prism in the presence of D_2O as the aqueous medium. Polarized light (s and p, corresponding to 0° and 90°, respectively) was used in the measurements. Lighter colors represent spectra obtained using p-polarized light (polarization set to 90°), while brighter colors represent spectra obtained using s-polarized light (polarization set to 0°).

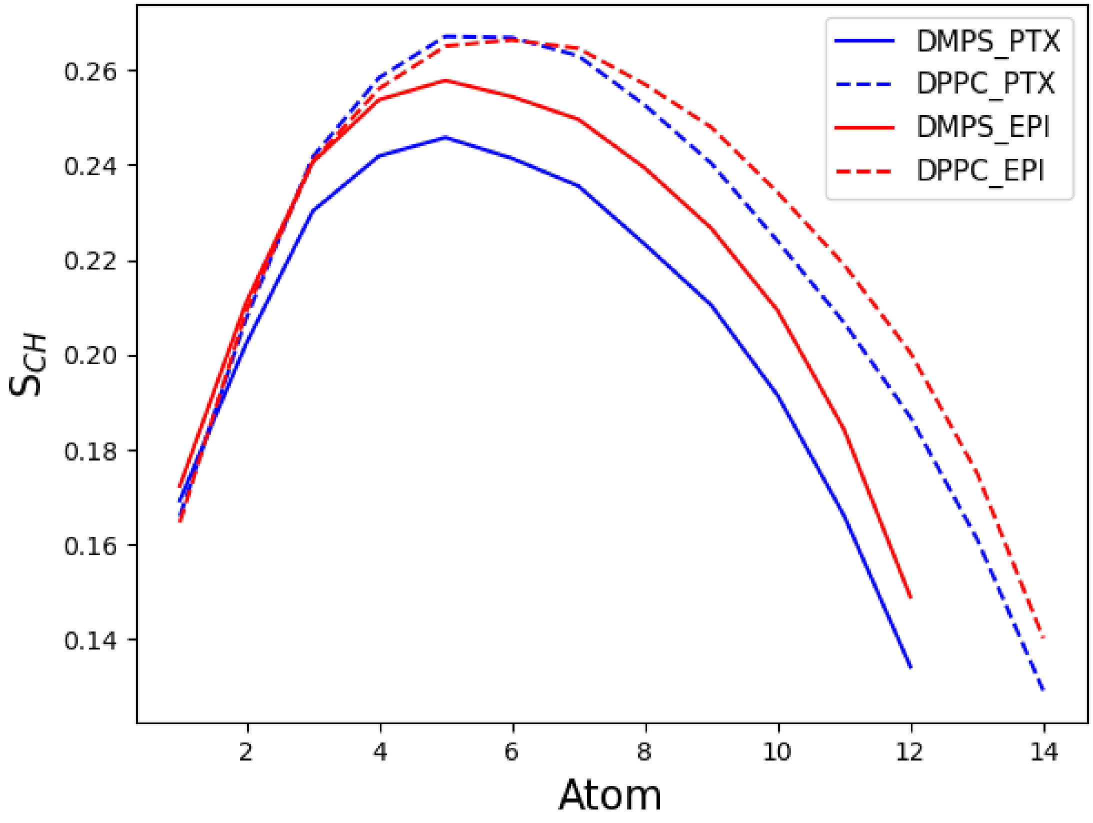

Figure S4. The acyl chain order parameter, S_{CH}, for the DMPS and DPPC calculated for monolayers containing single drug (EPI or PTX).

Table S3. The average number of H-bonds between molecules in pure monolayer.

	Number of H-bonds per molecule	
Molecule	CHOL	DMPS
CHOL	0.0002 ± 0.0001	0.1570 ± 0.0012
DMPS	0.1570 ± 0.0012	0.5287 ± 0.0024
DPPC	0.1393 ± 0.0008	0.1718 ± 0.0013

Table S4. The average number of H-bonds between molecules in single drug systems, containing a) EPI and b) PTX.

a)

	Number of H-bonds per molecule		
Molecule	CHOL	DMPS	EPI
CHOL	0.0004 ± 0.0001	0.1572 ± 0.0012	0.0481 ± 0.0018
DMPS	0.1572 ± 0.0012	0.4339 ± 0.0023	0.2693 ± 0.0054
DPPC	0.1467 ± 0.0008	0.1995 ± 0.0014	0.1645 ± 0.0042

b)

	Number of H-bonds per molecule		
Molecule	CHOL	DMPS	PTX
CHOL	0.0002 ± 0.0001	0.1554 ± 0.0012	0.0139 ± 0.0010
DMPS	0.1554 ± 0.0012	0.4681 ± 0.0023	0.2769 ± 0.0043
DPPC	0.1494 ± 0.0007	0.1902 ± 0.0013	0.2017 ± 0.0035

Table S5. The average number of H-bonds between molecules in drug mixture system.

	Number of H-bonds per molecule			
Molecule	CHOL	DMPS	EPI	PTX
CHOL	0.0008 ± 0.0001	0.1692 ± 0.0012	0.1014 ± 0.0028	0.0533 ± 0.0017
DMPS	0.1692 ± 0.0012	0.4377 ± 0.0026	0.2242 ± 0.0043	0.1996 ± 0.0038
DPPC	0.1509 ± 0.0008	0.2065 ± 0.0014	0.2971 ± 0.0048	0.2154 ± 0.0036

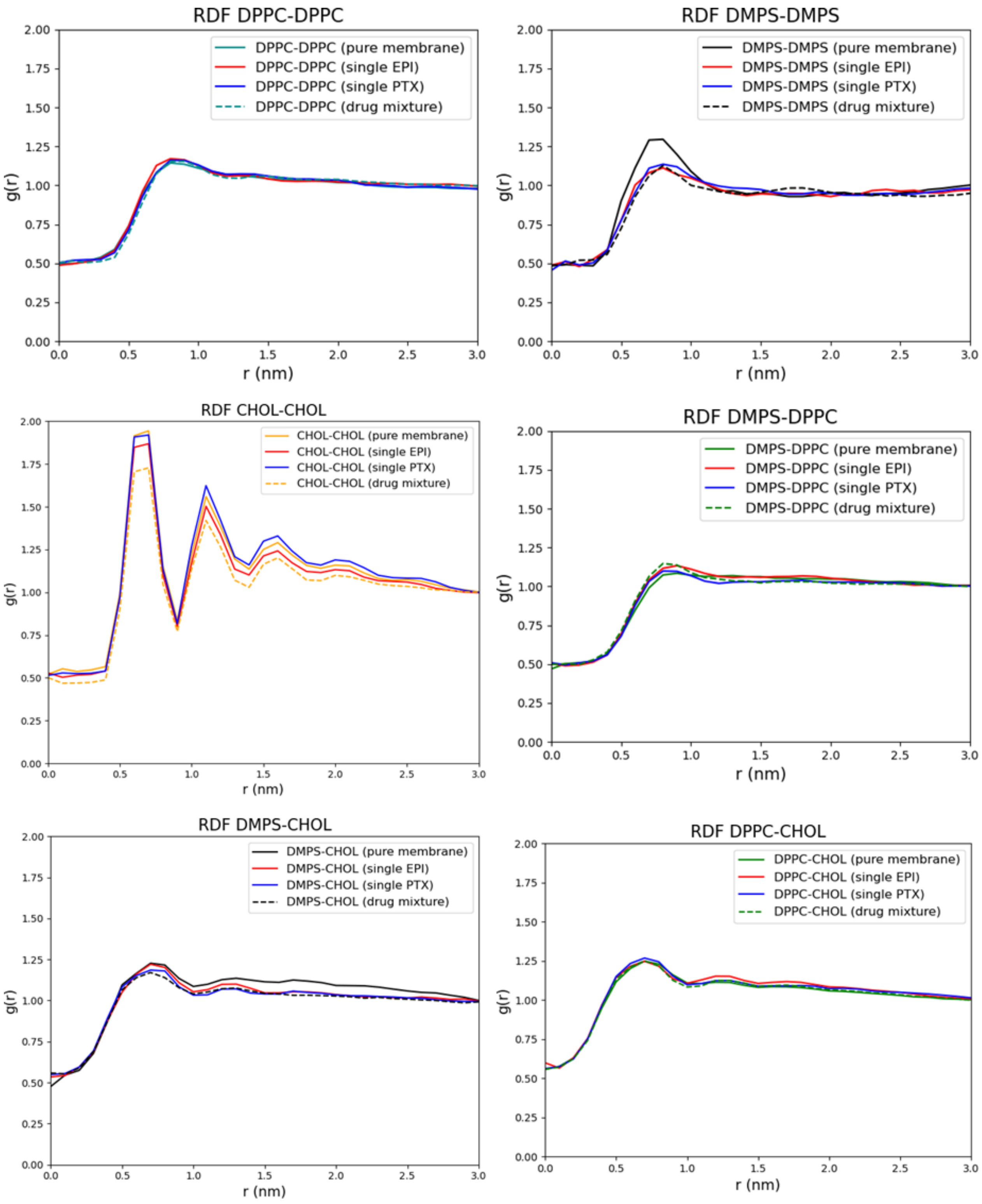

Figure S5. The radial distribution function between the monolayer components.
